# Impact of Physical Activity on Cellular Metabolism Across Both Neurodegenerative and General Neurological Conditions: A Narrative Review

**DOI:** 10.3390/cells13231940

**Published:** 2024-11-22

**Authors:** Vicente Javier Clemente-Suárez, Alejandro Rubio-Zarapuz, Pedro Belinchón-deMiguel, Ana Isabel Beltrán-Velasco, Alexandra Martín-Rodríguez, José Francisco Tornero-Aguilera

**Affiliations:** 1 Faculty of Sports Sciences, Universidad Europea de Madrid, Tajo Street, s/n, 28670 Madrid, Spain; vctxente@yahoo.es (V.J.C.-S.); alejandro.rubio@universidadeuropea.es (A.R.-Z.); josefrancisco.tornero@universidadeuropea.es (J.F.T.-A.); 2 Grupo de Investigación en Cultura, Educación y Sociedad, Universidad de la Costa, Barranquilla 080002, Colombia; 3 Department of Nursing, Faculty of Biomedical and Health Sciences, Universidad Europea de Madrid, 28670 Villaviciosa de Odón, Spain; pedro.belinchon@universidadeuropea.es; 4 Psychology Department, Faculty of life and natural sciences, Nebrija University, 28240 Madrid, Spain; abeltranv@nebrija.es; 5 Faculty of Applied Social Sciences and Communications, Universidad Internacional de la Empresa (UNIE), 28015 Madrid, Spain

**Keywords:** physical activity, cellular metabolism, exercise therapy, mitochondrial dysfunction, neurodegenerative diseases

## Abstract

Background: Regular physical activity plays a crucial role in modulating cellular metabolism and mitigating the progression of neurodegenerative diseases such as Alzheimer’s, Parkinson’s, and Multiple Sclerosis. Objective: The objective of this review is to evaluate the molecular mechanisms by which exercise influences cellular metabolism, with a focus on its potential as a therapeutic intervention for neurological disorders. Methods: A comprehensive literature review was conducted using peer-reviewed scientific articles, with a focus on the period between 2015 and 2024, to analyze the effects of exercise on mitochondrial function, oxidative stress, and metabolic health. Results: The findings indicate that exercise promotes mitochondrial biogenesis, enhances oxidative phosphorylation, and reduces reactive oxygen species, contributing to improved energy production and cellular resilience. These metabolic adaptations are associated with delayed disease progression and reduced symptoms in patients with neurodegenerative conditions. Additionally, integrating exercise with nutritional strategies may further enhance therapeutic outcomes by addressing metabolic disturbances comprehensively. Conclusions: This review concludes that personalized exercise protocols should be developed to optimize metabolic benefits for patients with neurological diseases, while future research should focus on biomarker development for individualized treatment approaches. These findings highlight the importance of non-pharmacological interventions in managing neurodegenerative diseases.

## 1. Introduction to Cell Metabolism and Its Importance

Regular physical activity is one of the most impactful non-pharmacological interventions for improving physiological health [1]. Exercise has been shown to positively affect multiple organ systems, primarily through its capacity to enhance cardiovascular, metabolic, and immune function. Aerobic exercise, for instance, improves heart health by increasing cardiac efficiency, reducing blood pressure, and promoting better circulation. Furthermore, physical activity can help prevent and manage chronic diseases such as diabetes, cancer, and osteoporosis by improving insulin sensitivity, enhancing immune responses, and promoting healthy weight maintenance [2]. On a cellular level, exercise triggers adaptations like mitochondrial biogenesis, which leads to increased energy production and better oxidative capacity of cells, improving overall metabolic health. Exercise-induced molecular changes in tissues also help reduce inflammation, improve tissue regeneration, and modulate fat metabolism, all of which contribute to the prevention of various diseases. For instance, regular physical activity has been associated with lower risks of breast and colon cancer, while also improving bone density, reducing the risk of fractures, and enhancing immune function [3].

**Figure 1:**
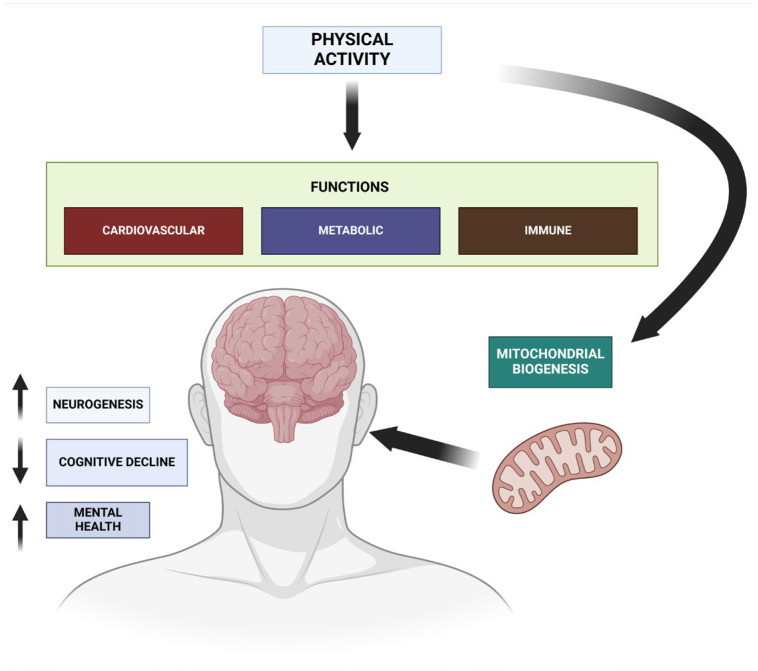
Physical activity impacts on mitochondria and consequently on brain health.

The physiological benefits of exercise also extend to mental health, where it positively influences brain function, helps alleviate depression, and reduces symptoms of anxiety, thanks to its effects on neurotransmitters and stress hormones (Figure 1) [4]. Regular physical activity stimulates the production of neurotransmitters such as serotonin and endorphins, which are associated with improved mood and reduced symptoms of depression and anxiety [5]. Exercise also lowers the levels of stress hormones like cortisol, further contributing to emotional balance and resilience against mental health disorders [6]. Furthermore, physical activity enhances cognitive function by promoting neurogenesis and increasing blood flow to the brain, which not only helps with mental well-being but also reduces the risk of cognitive decline [7]. Neurological diseases encompass any disorder affecting the nervous system, including conditions like epilepsy or stroke, with varied causes and courses. Neurodegenerative diseases are a subset of neurological diseases characterized by the progressive and irreversible loss of neurons, as seen in Alzheimer’s or Parkinson’s, leading to a gradual decline in cognitive and motor functions. Aging is associated with heightened susceptibility to significant diseases such as Alzheimer’s or Parkinson’s but also atherosclerosis, age-related macular degeneration, cataracts, and osteoporosis, which may share analogous underlying pathoetiologies [8]. All of these disorders entail oxidative stress, inflammation, and/or cellular apoptosis, instigated by cholesterol oxide derivatives, commonly referred to as oxysterols. These oxidized lipids originate either from the spontaneous and/or enzymatic oxidation of cholesterol on the steroid nucleus or on the side chain. Oxysterols’ capacity to cause significant dysfunction in organelles, particularly mitochondria, is crucial for RedOx homeostasis, inflammatory conditions, lipid metabolism, and the regulation of cell death, potentially elucidating their involvement in aging processes and age-related diseases [9]. Although it is believed that there is an absence of effective treatments for the majority of these diseases, which are anticipated to rise in prevalence due to increasing life expectancy and average age [10], physical activity plays a crucial role in managing them, helping to improve cellular resilience and slow disease progression. In Alzheimer’s disease, neurodegeneration is primarily driven by the accumulation of amyloid-beta plaques and tau protein tangles, which disrupt neuronal communication, trigger inflammation, and lead to cell death, especially in brain regions linked to memory and cognition [11]. Parkinson’s disease is characterized by the loss of dopaminergic neurons in the substantia nigra, leading to motor control deficits; mitochondrial dysfunction and oxidative stress play key roles in the progression of neuronal damage [12]. Multiple Sclerosis involves an autoimmune attack on the myelin sheath surrounding neurons, causing inflammation, demyelination, and eventual axonal damage, which disrupts nerve signal transmission [13]. Together, these neurobiological mechanisms underscore the cellular and metabolic vulnerabilities in each disease, highlighting the potential of exercise to support cellular resilience and mitigate neurodegenerative processes [14].

This study seeks to address the growing need for a deeper understanding of the relationship between physical activity, cellular metabolism, and neurological diseases. With rising incidences of neurodegenerative diseases like Alzheimer’s, Parkinson’s, and Multiple Sclerosis, there is a pressing need to explore non-pharmacological interventions that can mitigate disease progression and improve patient outcomes [15]. Regular physical exercise has been demonstrated to exert profound effects on mitochondrial function, oxidative stress reduction, and cellular metabolism, all of which are central to neurodegenerative disease pathology [14]. Therefore, the objective of this review was to evaluate the impact of physical activity on cell metabolism and its potential role in managing neurological diseases. This study aims to provide a comprehensive analysis of the molecular mechanisms by which exercise influences cellular processes, offering insights for developing effective therapeutic strategies to improve the quality of life for affected individuals.

### Material and Methods

The methodology for this narrative review was developed through a structured and systematic literature search, utilizing both primary and secondary academic sources. To ensure the inclusion of high-quality, peer-reviewed research, we accessed multiple scholarly databases, including PubMed, Science Direct, Scopus, and Web of Science. A combination of MeSH-compliant keywords was employed to capture the scope of our study on physical activity, mitochondrial function, neurodegenerative diseases, and cellular metabolism. The specific search terms included “cell metabolism”, “physical activity”, “mitochondrial biogenesis”, “Neurological disease”, and “exercise-induced molecular mechanisms”, allowing us to explore the role of exercise in mitigating metabolic dysfunction in neurodegenerative diseases. Our literature review encompassed articles published between January 2015 and October 2024, ensuring the inclusion of the most recent studies while also integrating seminal research from previous decades to provide a comprehensive understanding of the subject. This review was designed to focus on State-of-the-Art findings while ensuring historical context when necessary. This approach allowed us to trace the evolution of knowledge regarding mitochondrial dysfunction, oxidative stress, and energy metabolism-related neurodegenerative conditions.

To refine the selection of articles, we implemented rigorous exclusion criteria aimed at maintaining the review’s relevance and scientific integrity. The exclusion criteria were as follows: (i) studies that did not directly pertain to the core topics of the review—namely, the effects of physical activity on mitochondrial function and cellular metabolism in the context of neurodegenerative diseases, (ii) non-peer-reviewed or non-academic sources, such as dissertations, conference proceedings, and unpublished works, and (iii) articles published in languages other than English, unless a reliable translation was available and met the required quality standards. This filtering process ensured that the studies included in this review adhered to high methodological standards and focused on the specific aspects of mitochondrial dysfunction, cellular metabolism, and the relevant neuroprotective effects of exercise, focusing on specific neurological diseases impacted by cellular metabolism and physical activity. The primary conditions included “Alzheimer’s disease”, “Parkinson’s disease”, and “Multiple Sclerosis”. These terms served as key filters to identify relevant studies, allowing a targeted examination of how exercise and metabolic changes influence disease progression and therapeutic outcomes.

**Figure 2:**
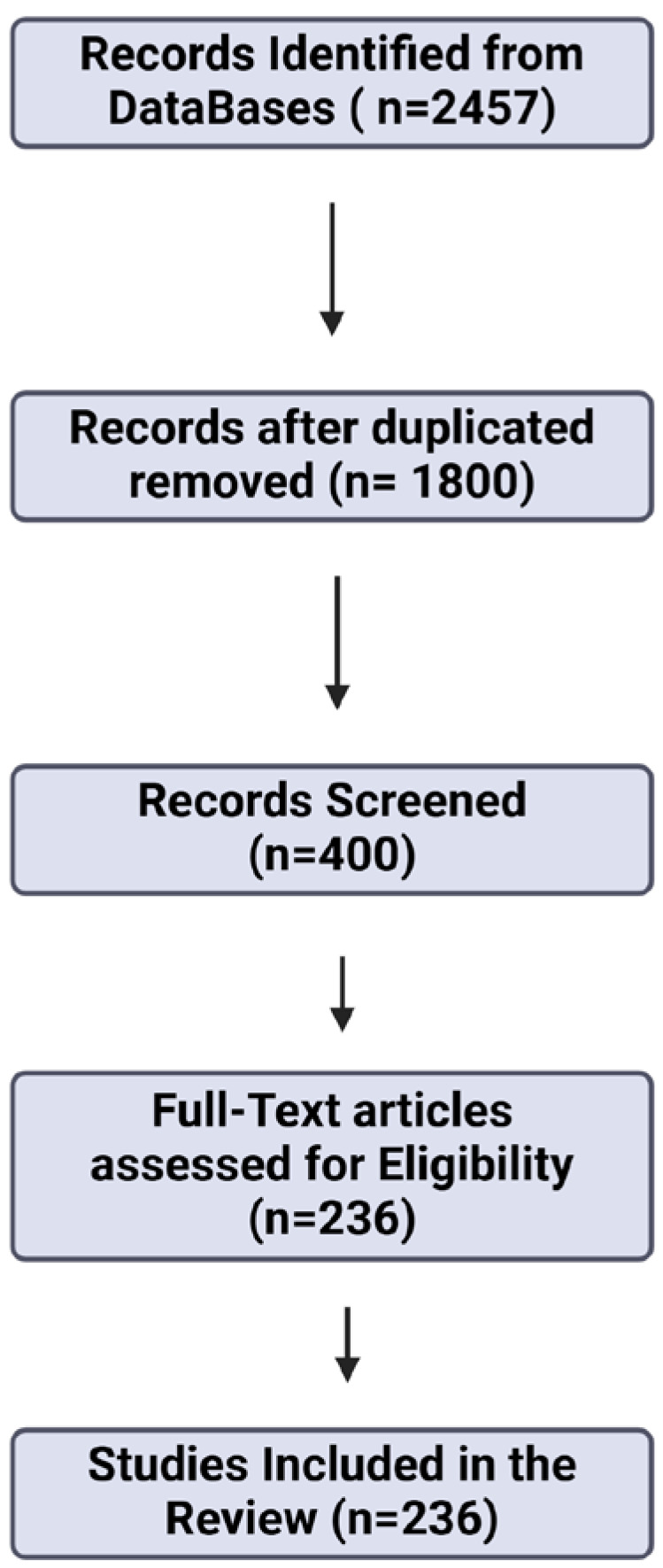
PRISMA flow diagram.

Once the exclusion criteria were applied, we screened the remaining articles based on their titles and abstracts to assess their alignment with the review’s objectives. Articles that appeared to fit the scope of this review underwent a full-text analysis to determine their methodological rigor and relevance. This detailed review resulted in the inclusion of 236 articles, which were critically examined and discussed by the authors. Each subtopic within the review, including the effects of exercise on mitochondrial biogenesis, oxidative stress regulation, and the pathophysiology of several neurodegenerative diseases and neurological conditions, was assigned to a group of authors based on their expertise. Through this collaborative approach, this review presents a multidisciplinary perspective on the interplay between physical activity, mitochondrial dysfunction, and neurodegenerative diseases. To further ensure the transparency and comprehensiveness of the review process, we utilized a PRISMA flow diagram (see Figure 2), which outlines the stages of article selection, from initial identification through final inclusion. This diagram provides a visual representation of the number of records identified, screened, and excluded, as well as the final studies incorporated into the review. It reflects the systematic methodology used to refine the pool of literature, ensuring that only studies of the highest quality and relevance were considered for inclusion in this narrative review.

This review aims to synthesize the current understanding of how mitochondrial dysfunction and impaired cellular metabolism contribute to neurological diseases and how physical activity may mitigate these effects through exercise-induced molecular mechanisms. By providing a thorough analysis of the literature, we aim to offer insights that are valuable to researchers, clinicians, and policymakers working in the fields of neurodegenerative diseases, metabolic health, and exercise physiology. Furthermore, this review emphasizes the potential of exercise as a therapeutic intervention, not only in managing the motor symptoms of neurological diseases but also in addressing the underlying metabolic disturbances that exacerbate disease progression.

## 2. General Effects of Physical Activity on Metabolism

The performance of physical activity has been demonstrated to confer a range of benefits on cellular metabolism, underscoring the importance of maintaining regular physical habits as a means of improving general well-being and maintaining optimal cellular metabolism. For instance, it has been observed that physical activity induces a number of adaptations in cellular metabolism, including an increase in the number of mitochondria and an improvement in their functional efficiency [16,17].

In this sense, physical exercise, particularly aerobic, serves as a potent stimulus that promotes mitochondrial biogenesis, a process whereby new mitochondria are generated within cells [18]. During physical activity, the demand for energy in muscle cells markedly increases, prompting various cellular adaptations to fulfill these energy requirements [19].

One of the primary mechanisms involved in this adaptation is the activation of AMPK (AMP-activated protein kinase) [20]. During exercise, the levels of AMP (adenosine monophosphate) in cells increase, which activates AMPK. This enzyme serves as an energy sensor, regulating the equilibrium between energy production and consumption. Upon activation, AMPK initiates a cascade of events aimed at activating PGC-1α (peroxisome proliferator-activated receptor gamma coactivator 1-alpha), a pivotal protein in the regulation of mitochondrial biogenesis [21].

PGC-1α is a transcriptional coactivator that regulates the expression of several genes involved in mitochondrial biogenesis [22]. Once activated, it increases the expression of transcription factors such as NRF-1 (nuclear respiratory factor 1) and NRF-2, which promote the expression of genes necessary for proper mitochondrial function [23]. Additionally, it activates TFAM (mitochondrial transcription factor A), which is essential for mitochondrial DNA transcription and mitochondrial replication. These mechanisms enable the cell to increase the number of mitochondria to meet the energy demand induced by physical activity, resulting in an enhanced capacity to produce ATP and improve cellular energy efficiency [24,25].

Conversely, mitochondrial efficiency is enhanced during exercise due to the production of adaptations in the components of the electron transport chain, which increases the activity of complex I (NADH oxidoreductase) and complex IV (cytochrome c oxidase). This action optimizes oxidative phosphorylation [26].

Another aspect of cellular metabolism that exhibits a marked improvement with physical activity is insulin sensitivity [27]. This process is crucial for maintaining the equilibrium of glucose levels within the body. It is established that insulin is a pivotal hormone that regulates the uptake of glucose by cells; thus, insulin sensitivity can be defined as the efficacy of cells in responding to this hormone [28,29].

During physical activity, particularly endurance and aerobic exercise, there is an increase in glucose uptake due to the elevated energy demand. This process enhances insulin sensitivity through mechanisms such as the translocation of glucose transporters [30]. Specifically, GLUT4 transporters are transported into the cell membrane [27]. These transporters are stored in vesicles inside the cell at rest. During exercise, a signaling process occurs that causes the migration of these vesicles towards the cell membrane, thereby favoring the entry of glucose into the cell. Although this process is independent of insulin during exercise, maintaining regular physical activity improves the response of these cells to insulin by increasing the amount of GLUT4 available for glucose transport when insulin binds to its receptor [31,32].

Furthermore, insulin signaling is augmented in muscle cells during physical activity. The binding of insulin to its receptor on the cell surface initiates an intracellular signaling cascade, including the phosphorylation of proteins such as IRS-1 (insulin receptor substrate-1) and the activation of PI3K (phosphoinositide 3-kinase) and Akt (protein kinase B), which facilitates the translocation of GLUT4 to the cell membrane and the uptake of glucose [33].

Conversely, the anti-inflammatory effect of physical activity is a significant contributor to enhanced insulin sensitivity. It has been established that chronic low-grade inflammation is linked to insulin resistance [34]. In this context, regular physical activity has been demonstrated to reduce the levels of inflammatory markers, including TNF-α (tumor necrosis factor-alpha) and IL-6 (interleukin-6), while increasing the release of anti-inflammatory myokines, such as IL-10. This improves insulin signaling and glucose uptake in muscle cells [35].

Furthermore, physical activity has been demonstrated to enhance hepatic insulin sensitivity. The liver plays a pivotal role in regulating glucose levels in the bloodstream, storing glucose in the form of glycogen and releasing it when necessary. Physical activity has been shown to increase glucose uptake by the liver, improve glycogen synthesis, and reduce hepatic glucose production [36].

Another beneficial effect of physical activity on cellular metabolism is the reduction in oxidative stress. Reactive oxygen species (ROS) are highly reactive molecules that can induce damage to lipids, proteins, and DNA, thereby contributing to the development of various pathologies and accelerating the aging process [37]. Regular exercise can attenuate these harmful effects by improving endogenous mechanisms and reducing excessive ROS production [38].

During exercise, there is an increase in reactive oxygen species (ROS) production due to elevated cellular oxygen consumption. However, the body adapts and strengthens its antioxidant systems through various mechanisms, including the increased expression and activity of endogenous antioxidant enzymes such as superoxide dismutase (SOD), glutathione peroxidase, and catalase [39]. These enzymes are essential for neutralizing ROS by transforming them into less reactive molecules and preventing cellular damage [40].

Regular physical activity has been demonstrated to enhance the expression of genes encoding these enzymes, thereby augmenting the cellular capacity to withstand oxidative stress. This gene regulation occurs via intracellular signaling pathways that are activated by physical exercise. One such pathway is the Nrf2 (nuclear factor erythroid 2-related factor 2) pathway, which controls the expression of numerous antioxidant genes. Conversely, the generation of ROS within the mitochondria is also diminished, thereby enhancing the efficiency of the electron transport chain [41]. Additionally, oxidative stress is mitigated by improving endothelial function and blood circulation, which, in turn, stimulates the production of nitric oxide (NO), a vasodilator molecule that improves blood flow and oxygenation in the tissues [42].

It has been demonstrated that physical activity induces the expression of heat shock proteins (HSPs), which have a protective role in cells. HSPs stabilize cells and repair damaged proteins, and they can interact with cellular components damaged by oxidative stress, facilitating their degradation and elimination [43].

In the context of lipid metabolism, the role of physical activity as an essential regulator is well documented, with effects on the mobilization of fats, their oxidation, and the regulation of blood lipid levels [44]. The triggering of enzymatic and hormonal adaptations by exercise improves the body’s lipid handling efficiency [45].

During endurance physical activity, the mobilization of fatty acids stored in adipocytes is increased. This process is mediated by the activation of lipolysis, which refers to the breakdown of triglycerides into free fatty acids and glycerol. The regulation of lipolysis is primarily dependent on the action of catecholamines, which are increased during exercise and bind to adrenergic receptors on adipocytes, thereby activating the enzyme hormone-sensitive lipase (HSL). This enzyme catalyzes the breakdown of stored triglycerides into free fatty acids, which are subsequently released into the bloodstream and transported to the muscles [46].

Once they reach the muscle cells, they are transported into the mitochondria, where they are oxidized to produce ATP through beta-oxidation, a metabolic process in which these fatty acids are degraded into acetyl-CoA units to generate energy in the Krebs cycle [47]. The practice of physical activity has been demonstrated to increase the expression and activity of enzymes involved in beta-oxidation [48]. These include carnitine palmitoyltransferase I (CPT-I), which facilitates the transport of fatty acids across the mitochondrial membrane, and acyl-CoA dehydrogenase, which catalyzes the first reaction of beta-oxidation [49].

Furthermore, aerobic physical exercise has been linked to a reduction in plasma triglyceride levels and an elevation in high-density lipoprotein (HDL) through mechanisms such as the activation of lipoprotein lipase-LPL, which is located on the surface of endothelial cells in capillaries, predominantly in skeletal muscle and adipose tissue. LPL facilitates the hydrolysis of triglycerides present in very low-density lipoproteins (VLDLs) and chylomicrons, resulting in the generation of free fatty acids, which can be subsequently taken up and utilized by tissues [50].

In contrast, physical activity has been demonstrated to exert a regulatory effect on lipid synthesis within the liver by decreasing the activity of the enzyme acetyl-CoA carboxylase (ACC), which is a crucial component in the synthesis of fatty acids [51]. This decreased activity results in a reduction in the production of malonyl-CoA, an inhibitor of carnitine palmitoyltransferase I (CPT-I). Low levels of malonyl-CoA promote the entry of fatty acids into the mitochondria for oxidation, thereby facilitating their utilization as an energy source. This metabolic change reduces fat accumulation in the liver, preventing hepatic steatosis [52].

It has been demonstrated that physical activity exerts an influence on protein metabolism, as it modulates protein synthesis and degradation in the body through a range of hormonal and molecular mechanisms that are responsive to the stimulus of physical activity. During anaerobic and endurance exercise, there is an increase in the demand for muscle proteins [53].

The mechanical stimulus of physical activity activates different signaling pathways to regulate protein synthesis, including the mTOR (mammalian target of rapamycin) pathway, which is one of the most important pathways. This pathway is activated in response to various stimuli, including the signaling of growth factors such as IGF-1 (insulin-like growth factor type 1), the availability of amino acids, and the mechanical activation of muscle fibers [54].

mTOR plays a pivotal role in protein synthesis by stimulating mRNA translation in ribosomes and enhancing the synthesis of new muscle proteins, which is a crucial process for muscle hypertrophy. Resistance exercise has also been demonstrated to be an effective method for activating this pathway, as it involves intense muscle contractions [55].

In addition to this pathway, physical exercise has been demonstrated to activate the MAPK (mitogen-activated protein kinase) pathway, which plays a role in the cellular response to stress and in the regulation of protein synthesis. This pathway is produced in response to muscle damage caused by exercise and has been shown to promote the repair of muscle tissue [56].

Furthermore, physical activity affects protein degradation, which is crucial for tissue regeneration. This process is primarily mediated by the ubiquitin–proteasome system and autophagy. Physical exercise has been demonstrated to reduce excessive protein degradation and promote equilibrium between protein synthesis and degradation.

Conversely, anabolic hormones, including growth hormones (GHs) and testosterone, are elevated in response to physical exercise, thereby promoting muscle hypertrophy [57]. These hormones activate signaling pathways that increase the translation and synthesis of new proteins. Additionally, levels of catabolic hormones, such as cortisol, are reduced, which facilitate the preservation of muscle proteins and favors an anabolic state [58].

## 3. Molecular Mechanisms

Physical activity exerts a profound and multifaceted influence on cellular metabolism through molecular mechanisms that are integral to the regulation of energy balance, mitochondrial function, and oxidative stress. These mechanisms form the backbone of the health benefits associated with regular exercise, which are particularly relevant in preventing and managing metabolic and neurological diseases [37]. At the core of these effects is AMP-activated protein kinase (AMPK), a key regulatory enzyme that functions as the cell’s energy sensor. AMPK activation is triggered by an increased ratio of AMP to ATP, a common occurrence during exercise when cellular energy demands rise significantly [59]. The activation of AMPK sets off a cascade of metabolic adjustments aimed at restoring energy balance within the cell. Specifically, AMPK stimulates catabolic pathways that generate ATP, such as glycolysis and fatty acid oxidation, while concurrently inhibiting anabolic processes, including protein and lipid synthesis, which are ATP-consuming activities [60]. This dual regulation not only ensures the cell meets its immediate energy needs during physical exertion but also initiates long-term adaptations that enhance metabolic efficiency. Among these adaptations is the increased translocation of glucose transporter type 4 (GLUT4) to the plasma membrane, which facilitates greater glucose uptake, and the upregulation of genes involved in fatty acid oxidation, both of which contribute to improved insulin sensitivity and overall metabolic health [61]. These metabolic shifts underscore the pivotal role of AMPK in mediating the beneficial effects of physical activity, highlighting its importance in both energy homeostasis and disease prevention.

In addition to its role in energy balance regulation, physical activity significantly influences mitochondrial function, primarily through the stimulation of mitochondrial biogenesis. This process is driven by the transcriptional coactivator peroxisome proliferator-activated receptor gamma coactivator 1-alpha (PGC-1α), a master regulator of mitochondrial gene expression [62]. PGC-1α is upregulated in response to several exercise-induced signaling pathways, including AMPK, p38 mitogen-activated protein kinase (p38 MAPK), and calcium/calmodulin-dependent protein kinase (CaMK). The activation of PGC-1α leads to the coordinated expression of nuclear and mitochondrial genes involved in oxidative phosphorylation, resulting in an increase in both the quantity and functionality of mitochondria within cells [63]. This enhanced mitochondrial biogenesis is critical for maintaining energy production, especially in tissues with high metabolic demands such as skeletal muscle and the brain. Furthermore, exercise promotes mitochondrial dynamics by stimulating both fusion and fission processes, which are essential for the maintenance of mitochondrial integrity and function. Mitochondrial fusion helps to mitigate the effects of mitochondrial DNA mutations by mixing the contents of partially damaged mitochondria, thereby maintaining mitochondrial function, while fission allows for the removal of damaged mitochondria through mitophagy [64]. This dynamic remodeling of the mitochondrial network not only supports cellular energy production but also enhances the cell’s ability to adapt to metabolic stress, making it a vital component of the cellular response to physical activity [65].

Furthermore, the role of PGC-1α in exercise-induced mitochondrial adaptations extends beyond mere energy production. This coactivator also plays a crucial role in the regulation of oxidative metabolism, particularly in terms of balancing the production of reactive oxygen species (ROS) and maintaining cellular redox homeostasis [10]. PGC-1α stimulates the expression of various antioxidant enzymes, including superoxide dismutase (SOD) and glutathione peroxidase, which are essential for neutralizing ROS. By enhancing the antioxidant capacity of the cell, PGC-1α not only protects against oxidative damage but also ensures that ROS production during exercise remains within a physiologically beneficial range [66]. This protective mechanism is particularly important in tissues like skeletal muscle, where high levels of ROS are generated during intense physical activity. The ability of PGC-1α to modulate both mitochondrial biogenesis and antioxidant defense underscores its pivotal role in the cellular adaptations to exercise, which contribute to improved metabolic health and reduced susceptibility to oxidative stress-related diseases [67].

Moreover, ROS, which are byproducts of metabolic processes, play a complex role in cellular metabolism, particularly in the context of physical activity. Exercise-induced ROS production is a double-edged sword; on one hand, excessive ROS can lead to oxidative damage to proteins, lipids, and DNA, contributing to cellular dysfunction and aging [68]. On the other hand, moderate levels of ROS generated during exercise serve as crucial signaling molecules that trigger adaptive responses aimed at enhancing cellular resilience [67]. These ROS-mediated signaling pathways include the activation of nuclear factor erythroid 2–related factor 2 (Nrf2), a transcription factor that regulates the expression of antioxidant defense genes, and mitogen-activated protein kinases (MAPKs), which are involved in various cellular processes including inflammation, cell differentiation, and apoptosis [69]. Through these pathways, ROS act as signaling entities that stimulate the upregulation of endogenous antioxidant systems, thereby reducing oxidative stress and enhancing mitochondrial function. This process highlights the importance of controlled ROS production during exercise. By modulating redox-sensitive signaling pathways, physical activity not only protects cells from oxidative damage but also promotes a metabolic environment that is conductive to long-term cellular health and function. This adaptive response is particularly important in preventing metabolic dysfunction and neurodegenerative diseases, where oxidative stress plays a significant pathological role [70,71].

The interplay between AMPK activation, PGC-1α-mediated mitochondrial biogenesis, and ROS signaling reflects the sophisticated nature of the cellular response to physical activity. Each of these mechanisms is interconnected, forming a comprehensive network that ensures cells can meet the increased energy demands of exercise while simultaneously protecting against metabolic stress [72]. For example, the activation of AMPK not only enhances energy production but also indirectly supports mitochondrial health by upregulating PGC-1α. Similarly, the controlled production of ROS during exercise acts as both a signal for mitochondrial biogenesis and a trigger for antioxidant defenses, ensuring that cells remain resilient in the face of oxidative stress. The combined effect of these pathways is a more robust and adaptable cellular metabolism, which is better equipped to handle the demands of both exercise and everyday metabolic challenges. Highlighting the importance of a holistic approach to understanding how physical activity influences cellular health, particularly in the context of preventing and managing diseases associated with metabolic dysfunction [73].

Together, these molecular mechanisms underscore the critical role of physical activity in modulating cellular metabolism, contributing to a stronger metabolic profile. These adaptations are essential not only for maintaining metabolic health but also for mitigating the risk of neurodegenerative diseases, which are increasingly recognized as being linked to metabolic dysfunction. The interplay between these pathways underscores the deep impact of physical activity on cellular function, emphasizing its potential as a therapeutic intervention for a wide range of metabolic and neurological conditions.

## 4. Relationship Between Cell Metabolism and Neurological Diseases

The relationship between cell metabolism and neurological diseases is increasingly becoming a focal point in understanding the pathogenesis of neurodegenerative disorders. Therefore, metabolic dysfunction is now recognized but also as a precursor of neurodegeneration, contributing significantly to the onset and progression of diseases such as Alzheimer’s disease, Parkinson’s disease, and Multiple Sclerosis [74]. At a cellular level, metabolic disturbances manifest through several interconnected processes, including mitochondrial dysfunction, impaired energy metabolism, and elevated oxidative stress, leading to neuronal damage. These disturbances interfere in the delicate energy balance within neurons and glial cells, fostering the conditions for cellular impairments and, ultimately, neurodegeneration. The recognition of these metabolic abnormalities as early indicators of neurodegeneration has opened new avenues for research showcasing the urgent need to explore how these cellular processes contribute to disease pathophysiology and how they might be effectively targeted for therapeutic intervention [75].

**Figure 3:**
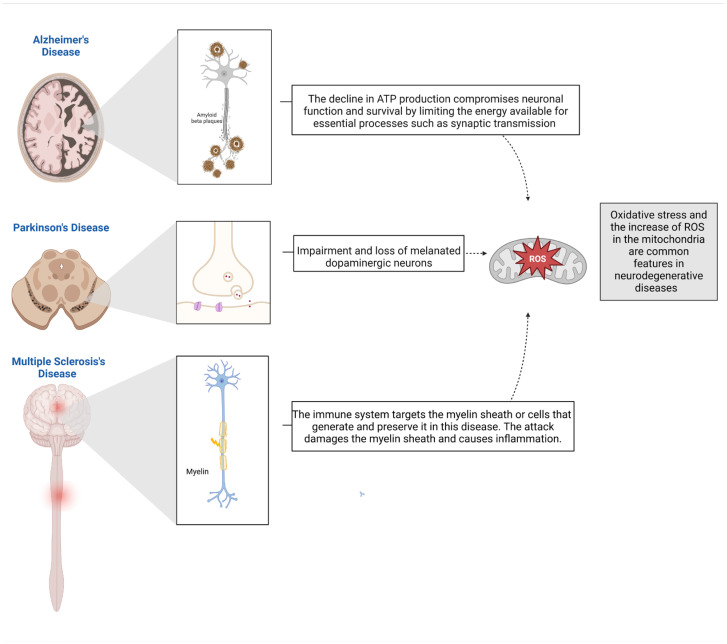
Conditions that neurological diseases present due to ROS.

Mitochondria are critical for maintaining neuronal energy metabolism. Neurons, due to their high energy demands and limited capacity for glycolysis, are particularly vulnerable to mitochondrial dysfunction [76]. In Alzheimer’s, mitochondrial dysfunction is characterized by a marked reduction in ATP production, an increase in the generation of ROS, and impaired calcium homeostasis (Figure 3). These mitochondrial alterations are not merely byproducts of the disease but are increasingly thought to actively contribute to its progression. For instance, the decline in ATP production compromises neuronal function and survival by limiting the energy available for essential processes such as synaptic transmission and the maintenance of ionic gradients [77]. Simultaneously, the overproduction of ROS leads to oxidative damage to cellular components, including lipids, proteins, and DNA, which exacerbates amyloid-beta toxicity and tau hyperphosphorylation—two hallmark features of Alzheimer’s disease pathology. This oxidative stress, coupled with disrupted calcium signaling within mitochondria, further accelerates neuronal death, creating a vicious cycle of neurodegeneration that progressively impairs cognitive function [78]. Moreover, the accumulation of dysfunctional mitochondria in neurons has been linked to the failure of mitophagy, the process by which damaged mitochondria are removed from the cell. This accumulation exacerbates cellular stress and promotes the aggregation of toxic proteins, contributing to the neuronal degeneration observed in Alzheimer’s disease. Additionally, the interaction between dysfunctional mitochondria and the endoplasmic reticulum (ER) leads to the disruption of ER-mitochondrial signaling pathways, eliciting further metabolic disturbances and stress responses that accelerate the neurodegenerative process, highlighting the complexity of mitochondrial involvement in this disease [11].

The deterioration of dopaminergic neurons in the brain is believed to be a critical factor in the onset of Parkinson’s disease. These neurons are susceptible to degeneration due to their extensive branching and the significant energy demands necessary for transmitting nerve signals throughout this elaborate network. Similarly, mitochondrial dysfunction plays a main role in the pathogenesis of Parkinson’s disease, where it is closely linked to genetic mutations that affect mitochondrial quality control. Mutations in genes such as PINK1 and Parkin, which are involved in mitochondrial autophagy (mitophagy), lead to the accumulation of damaged mitochondria in dopaminergic neurons, which is particularly detrimental given the high metabolic demands of these neurons and their susceptibility to oxidative stress [79]. Dopaminergic neurons, responsible for the production of dopamine (the neurotransmitter involved in motor control) are particularly sensitive to changes in mitochondrial function, resulting in increased ROS production, further damaging cellular structures and contributing to the selective vulnerability of dopaminergic neurons in Parkinson’s disease (Figure 3). The loss of these neurons in the substantia nigra, which is a defining feature of Parkinson’s disease, leads to characteristic motor symptoms, such as tremors, rigidity, and bradykinesia. Furthermore, the role of mitochondrial dysfunction in Parkinson’s disease extends beyond the direct effects on neuronal survival. Dysfunctional mitochondria can also impair synaptic function by disrupting the supply of ATP required for neurotransmitter release and synaptic vesicle recycling. This impairment exacerbates the communication breakdown between neurons, contributing to the progressive nature of the disease [12]. Additionally, the oxidative stress generated by dysfunctional mitochondria can lead to the oxidation of dopamine itself, creating toxic byproducts that further damage neurons. This cascade of events highlights the multidimensional impact of mitochondrial dysfunction in this disease, making it a critical target for potential therapeutic interventions aimed at preserving mitochondrial function and preventing neuronal loss [80].

In Multiple Sclerosis, the most often occurring demyelinating disorder of the central nervous system, the immune system targets the myelin sheath or cells that generate and preserve it in this disease. The attack damages the myelin sheath and causes swelling—also known as inflammation. Also, there has been evidence of numerous characteristics of an inflammatory autoimmune disorder, including the disruption of the blood–brain barrier (BBB). The blood–brain barrier (BBB) is a sophisticated structure composed of cerebral endothelial cells, pericytes, and their basal lamina, which is encased and supported by astrocytes and perivascular macrophages [81]. Thus, mitochondrial dysfunction is also a significant contributor to the neurodegenerative processes that characterize the disease [82]. Recent studies, on the other hand, have shed light on the role that mitochondrial dysfunctions play in driving these pathological processes to a greater degree. In particular, reactive oxygen species (ROS), which, when produced in excessive amounts, can result in oxidative stress, have been associated with demyelination and axonal damage as mediators. Macrophages and their companions in the central nervous system are among the cell populations that have been investigated the most in the context of ROS-mediated tissue damage in multiple sclerosis (Figure 3) [80].

This finding affects mitochondrial DNA and oxidative phosphorylation, impairing the energy production necessary for the repair and maintenance of neurons and glial cells, which are crucial for the function of the CNS. This energy deficit, particularly in the context of demyelinated axons, leads to axonal degeneration, a key factor in the progression of Multiple Sclerosis [83]. Moreover, the increased production of ROS in mitochondria contributes to oxidative stress, which not only damages neuronal cells but also perpetuates the inflammatory environment within the CNS, further driving disease progression [84]. Further on, the energy deficit caused by mitochondrial dysfunction is particularly problematic as demyelinated axons require more energy to conduct nerve impulses. The impaired ability of mitochondria to meet these increased energy demands results in axonal degeneration and contributes to the chronic disability seen in Multiple Sclerosis patients. Additionally, the inflammatory environment in this disease further exacerbates mitochondrial dysfunction, creating a feedback loop where inflammation and mitochondrial damage reinforce each other, accelerating the progression of the disease. This interplay between immune-mediated damage and metabolic dysfunction highlights the complexity of the pathology of Multiple Sclerosis and underscores the importance of targeting mitochondrial health in therapeutic strategies [85].

The interplay between metabolic dysfunction and inflammation is another critical aspect of the relationship between cell metabolism and neurological diseases, characterized by chronic, low-grade inflammation linked to metabolic disturbances such as insulin resistance, obesity, and dyslipidemia. This state of systemic inflammation can exacerbate neuroinflammation, a key driver of neurodegeneration in diseases like Alzheimer’s disease, Parkinson’s disease, and Multiple Sclerosis [86]. For instance, in Alzheimer’s disease, metabolic inflammation is associated with insulin resistance in the brain, which leads to impaired glucose metabolism and the subsequent accumulation of toxic amyloid-beta plaques and hyperphosphorylated tau proteins, which contributes to the neuronal dysfunction and cell death that characterizes this disease, bringing attention to the link between metabolic health and brain function. Moreover, in Parkinson’s disease, systemic inflammation and oxidative stress are similarly implicated in the degeneration of dopaminergic neurons. Chronic inflammation, often driven by metabolic factors, contributes to the oxidative damage of these neurons, exacerbating the progression of the disease [87]. Furthermore, metabolic disturbances such as dyslipidemia and insulin resistance have been shown to influence the pathophysiology of Parkinson’s disease, potentially offering new targets for intervention. In Multiple Sclerosis, the role of inflammation is particularly pronounced, as metabolic inflammation not only contributes to neurodegeneration but also exacerbates the autoimmune response against myelin. This dual role of inflammation and metabolic dysfunction underscores the complexity of Multiple Sclerosis and the potential for targeting these pathways in treatment [88]. In addition to these mechanisms, the role of metabolic hormones such as leptin, adiponectin, and ghrelin in neurological diseases is gaining attention. These hormones, involved in the regulation of energy balance, appetite, and glucose metabolism, are shown to influence brain function and are implicated in the pathogenesis of neurodegenerative diseases. For example, leptin, which is produced by adipose tissue, has neuroprotective effects and can enhance synaptic plasticity. However, in conditions of leptin resistance, such as obesity, these protective effects are diminished, potentially contributing to the development of Alzheimer’s disease. Similarly, adiponectin, an anti-inflammatory hormone, is found at lower levels in individuals with metabolic syndrome, which may exacerbate neuroinflammation and oxidative stress in neurodegenerative diseases. Understanding the role of these metabolic hormones in brain health may provide new insights into the links between systemic metabolism and neurological disease as well as open new paths for treatment [89,90].

The relationship between cell metabolism and neurological diseases is complex, with metabolic dysfunction playing a central role in the pathogenesis of neurodegenerative disorders [91]. Mitochondrial dysfunction, impaired energy metabolism, and inflammation are common characteristics of diseases such as Alzheimer’s disease, Parkinson’s disease, and Multiple Sclerosis. Understanding these relationships at a deeper level is crucial for developing new therapeutic approaches that target the metabolic foundations of these diseases, offering hope for more effective treatments and improved patient outcomes [25]. As research continues to uncover the intricate connections between metabolism and neurodegeneration, there is a growing potential to identify metabolic biomarkers for early diagnosis and to develop interventions that can modify the disease course by restoring metabolic health.

## 5. Clinical and Preclinical Evidence

At present, there is a substantial body of clinical and preclinical evidence elucidating the influence of physical activity on cellular metabolism. Preclinical evidence has been pivotal in elucidating the molecular and cellular mechanisms that emerge in response to physical exercise.

In vivo animal models, predominantly murine, have permitted the implementation of rigorous investigations examining the impact of physical activity on cellular biology. One of the inaugural investigations in this field was conducted by Baar and Esser (1999) with regard to mitochondrial biogenesis. This study demonstrated that endurance training augments the activity of the mTOR pathway, thereby stimulating the synthesis of mitochondrial proteins and the formation of new mitochondria in skeletal muscle [92].

Another study, conducted in 2006 by Hood et al., demonstrated that physical activity resulted in an increase in the expression of PGC-1α in the skeletal muscles of mice, leading to an enhancement in the quantity and functionality of mitochondria. Furthermore, the oxidative capacity of muscle cells and ATP production were enhanced [93].

More recent research demonstrated that exercise in mice significantly increases the expression of PGC-1α and other regulators of mitochondrial biogenesis, such as NRF1 and TFAM. This effect suggests that cellular energy efficiency is also improved [94]. Recent clinical studies, such as the one conducted by Robinson et al. (2017) in older adults, have demonstrated that resistance training increases the expression of genes related to mitochondrial biogenesis in skeletal muscle, including PGC-1α. Participants exhibited a significant increase in mitochondrial content and respiratory capacity of muscle mitochondria, indicating that exercise may mitigate the effects of aging on mitochondrial function [16].

In another clinical study, Granata et al. (2018) investigated the impact of high-intensity interval training (HIIT) on mitochondrial biogenesis in a cohort of healthy participants. The findings indicated that the training regimen, when administered for a period of six weeks, led to a notable elevation in the expression of PGC-1α, NRF1, and TFAM in skeletal muscle. Additionally, there was a discernible enhancement in the respiratory capacity of muscle mitochondria and mitochondrial protein content [95].

A series of studies conducted on mice with high-fat diet-induced obesity demonstrated that regular physical activity enhances insulin signaling and glucose uptake in both muscle and liver tissues. Consequently, Morino et al. (2006) revealed that exercise in mice elevates the phosphorylation of IRS-1 and Akt, pivotal elements in the insulin signaling cascade. Additionally, intramuscular and hepatic lipid accumulation was diminished, thereby enhancing insulin sensitivity [96].

A study conducted by Lessard et al. (2011) corroborated these findings, demonstrating that regular exercise in a mouse model with low aerobic capacity is capable of restoring metabolic function and insulin sensitivity [97]. Moreover, Stanford et al. (2015) observed that exercise enhances the phosphorylation of Akt and AMPK, thereby improving glucose uptake and reducing intramuscular lipid accumulation [98].

Malin et al. (2016) conducted a clinical study in overweight and obese adults. The results showed that different types of exercise (resistance, aerobic, and combined) significantly improved insulin sensitivity, which was measured by intravenous glucose tolerance. Reductions in visceral fat and improvements in body composition were also observed [99]. Another study by Solomon et al. in 2018 in adult patients with prediabetes evaluated the impact of aerobic exercise for 12 weeks. The results showed that this training significantly improved insulin sensitivity, increasing the rate of glucose uptake by 25% compared to the control group. There were also significant reductions in visceral fat and systemic inflammation levels, which were measured by C-reactive protein (CRP) concentrations [100].

Preclinical studies have also evaluated the impact of physical activity on the reduction in oxidative stress. One of the first studies conducted in a rat model showed that exercise increases the activity of antioxidant enzymes such as SOD and catalase, protecting cells from oxidative damage by neutralizing reactive oxygen species. (Powers et al., 1994). Merry and Ristow (2016) showed that exercise in mice increases the expression of these enzymes and found a decrease in the levels of oxidative damage markers such as lipid peroxidation and protein oxidation [101,102].

Clinical studies, such as the one conducted in 2015 by Gomez-Cabrera et al. in young and older adults, showed the impact of aerobic exercise on oxidative stress markers and antioxidant capacity. The results of this study showed that the antioxidant enzyme activity was significantly increased, and the levels of biomarkers of oxidative damage were reduced. Moreover, the impact was more significant in older participants, suggesting that exercise may be especially beneficial in attenuating the increased oxidative stress associated with aging [103].

In another clinical study conducted by Mason et al. (2016), the effects of aerobic exercise on oxidative stress reduction in older adults were evaluated. The results demonstrated a significant reduction in malondialdehyde (MDA) levels, as well as an increase in the activity of the antioxidant enzymes SOD and glutathione peroxidase, in comparison to the control group. Furthermore, the exercising individuals exhibited an improvement in plasma total antioxidant capacity [104].

In regard to lipid metabolism, a study conducted by Riechman et al. (2004) demonstrated that physical activity in mice resulted in a reduction in plasma triglyceride levels and an increase in high-density lipoprotein (HDL) levels, thereby improving the blood lipid profile [105]. These findings were supported by additional research, which also demonstrated that the activity of acetyl-CoA carboxylase (ACC) in the liver is reduced, which, in turn, leads to a reduction in the production of fatty acids and a reduction in the accumulation of fat in the liver [106].

A more recent study conducted in a mouse model demonstrated that exercise increases lipoprotein lipase (LPL) activity in skeletal muscle, thereby improving triglyceride clearance from the circulation and decreasing acetyl-CoA carboxylase (ACC) activity in the liver [107].

Clinical studies have demonstrated that physical activity effectively regulates lipid metabolism in humans. A 2019 study by Kraus et al. in adults with dyslipidemia evaluated the effects of aerobic and resistance exercise on blood lipid levels. The results indicated that moderate-to-intense aerobic exercise significantly reduced triglyceride levels and increased high-density lipoprotein levels. Resistance training was also found to improve body composition, decreasing fat mass and increasing lean mass [72].

In this line of inquiry, a study conducted by Korpelainen et al. (2019) evaluated the impact of combined aerobic-endurance exercise on the regulation of lipid metabolism in overweight and obese adults [108]. The findings indicated that this combination resulted in a notable reduction in triglyceride and LDL-cholesterol levels in comparison to the control group. Additionally, there was an enhancement in body composition and a modification in the lipid profile, accompanied by an increase in lipoprotein lipase activity and a decrease in acetyl-CoA carboxylase (ACC) activity in adipose tissue and skeletal muscle [109].

Conversely, the preclinical evidence on the impact of physical activity on neurological diseases permits an understanding of the molecular and cellular mechanisms underlying the benefits of exercise. These studies, primarily conducted in rodents, have consistently demonstrated protective and therapeutic effects in various neurological pathologies, including Alzheimer’s Disease (AD), Parkinson’s Disease (PD), Multiple Sclerosis (MS), and Stroke.

A study conducted by Nichol et al. (2007) in a murine model of AD demonstrated that physical exercise can mitigate the accumulation of amyloid plaques and phosphorylated tau in the brain. Moreover, there was an increase in neurogenesis and synaptic plasticity in the hippocampus, which led to enhanced cognitive performance and a reduction in brain inflammation and oxidative stress [110]. In a further study conducted by Andreotti et al. (2020), the impact of physical activity on cognitive function was evaluated in a transgenic mouse model of AD. The results demonstrated a significant reduction in amyloid plaques in the hippocampus and cerebral cortex. Additionally, there was an increase in the expression of the brain-derived neurotrophic factor (BDNF) and other synaptic proteins, which improved synaptic plasticity and neurogenesis in the hippocampus [111].

The results of clinical studies lend support to the positive outcomes observed with respect to cognitive function and disease progression. In a 2022 study, Baker and colleagues evaluated the impact of aerobic exercise on patients with mild-to-moderate AD. The results demonstrated significant improvements in memory and attention, accompanied by reductions in disease biomarkers such as tau protein and amyloid plaques [112]. Additionally, a longitudinal study by Pfeifer and colleagues (2022) indicated that regular physical activity resulted in a lower accumulation of disease-associated biomarkers compared to controls, suggesting a preventive role in the progression of AD [113].

A study conducted by Tajiri et al. (2010) addressed the impact of physical activity in a mouse model of induced PD. The researchers observed that exercise increased the density of dopaminergic terminals and ameliorated motor deficits, indicating a neuroprotective effect [114]. In 2021, Paillard et al. corroborated these findings in a mouse model of PD induced by the administration of 6-hydroxydopamine (6-OHDA). The study demonstrated that physical exercise markedly elevated BDNF levels in the striatum and hippocampus, in addition to an increased density of dopaminergic neurons in the substantia nigra and striatum, compared to sedentary mice [115].

A recent clinical study evaluated the impact of high-intensity interval training (HIIT) on patients with early-stage PD. The results demonstrated that participants who underwent this training exhibited notable enhancements in motor symptoms, walking speed, and balance when compared to the control group [116]. In 2021, a study conducted by Petzinger et al. addressed the impact of aerobic exercise on cognitive function in PD. The results demonstrated that the exercise group experienced significant improvements in memory, attention, and executive function compared to the control group. This finding suggests that this type of exercise not only improves motor symptoms but also the cognitive function associated with this pathology [117].

In regard to MS, physical activity has demonstrated advantageous effects in animal models through the modulation of the immune system, which has been observed to result in a reduction in proinflammatory T cell infiltration into the central nervous system. In a study conducted by Klopstein and colleagues in 2016, the impact of physical exercise on inflammatory processes associated with MS was investigated in a mouse model. The findings indicated a notable reduction in neuroinflammation and an improvement in clinical symptoms in mice with experimental autoimmune encephalomyelitis (EAE). Another study conducted by Proschinger et al. [118] on a murine model of MS demonstrated that exercise resulted in a reduction in demyelinating lesions and an improvement in motor abilities when compared to control mice [42,118].

A clinical study conducted by Najafi et al. [119] corroborated the preclinical findings. The objective of this study was to evaluate the impact of physical activity on the quality of life of patients with MS. The findings indicated that exercise resulted in a reduction in the severity of associated clinical symptoms and a decline in markers of inflammation and demyelination [119]. These findings have been corroborated by other studies. In a separate study, Briken et al. [120] investigated the impact of resistance training on the functional capacity and muscle strength of patients with MS. The results demonstrated notable enhancements in walking velocity, muscle strength, and stair climbing capability. Furthermore, a notable decline in fatigue levels and quality of life was observed. Other authors investigated the potential benefits of aerobic exercise on the quality of life and fatigue levels of MS patients. They utilized the Multiple Sclerosis Quality of Life-54 (MSQOL-54) scale to assess quality of life and the Fatigue Scale for Motor and Cognitive Functions (FSMC) to evaluate fatigue. Their findings indicated that the exercise group exhibited a notable reduction in fatigue and an improvement in quality of life compared to the control group [120].

In addition, physical activity has been demonstrated to exert beneficial effects in the context of stroke. A study conducted by Zhang et al. [121] demonstrated that post-stroke exercise in a rat model improved motor recovery and increased cell proliferation in the hippocampus, thereby facilitating the repair of damaged brain tissue [121]. Zheng et al. conducted another study to evaluate the impact of physical activity on functional recovery and neuroplasticity after an induced stroke in a rat model. The results demonstrated a significant recovery of motor activity, an increase in the expression of proteins associated with neuroplasticity, such as the BDNF, as well as an increase in the synthesis of synaptophysin in the affected areas of the brain. Additionally, there was an increase in angiogenesis in damaged brain tissue and a reduction in neuronal apoptosis [122,123].

The results of clinical studies conducted in accordance with these parameters have corroborated the findings of the preclinical studies. A study conducted by Moore et al. (2021) examined the influence of aerobic exercise on motor recovery and fatigue reduction in individuals who have experienced a stroke. Following a 12-week period, the findings revealed notable enhancements in motor function, as assessed by the Fugl-Meyer scale, and a reduction in fatigue when compared to the control group. Furthermore, an improvement in quality of life was observed, as evaluated by the stroke-specific quality of life questionnaire (SS-Qol-Stroke Specific Quality of Life Scale) [124].

A study conducted by Eng et al. (2020) evaluated the impact of resistance training on muscle strength and functional performance in stroke survivors. After 12 weeks of training, significant improvements were observed in muscle strength, walking speed, and the ability to perform daily activities, compared to the control group. These effects were maintained at least 3 months after the intervention [125].

In another clinical trial conducted in 2020 by Koch et al., the efficacy of an aerobic and resistance exercise program for the functional recovery of stroke patients was evaluated. The results demonstrated significant improvements in functional capacity, as assessed with the Barthel Functional Recovery Scale, as well as improvements in mobility. Furthermore, a reduction in depression levels and an improvement in quality of life were observed [126].

## 6. Cell Metabolism in Alzheimer’s Disease

Alzheimer’s disease (AD) is one of the most prevalent neurodegenerative conditions worldwide, characterized by progressive cognitive decline, memory impairment, and widespread neuronal dysfunction. Beyond the classical pathological markers of amyloid-beta (Aβ) plaques and tau protein hyperphosphorylation, increasing evidence points to mitochondrial dysfunction and disrupted cellular metabolism as central factors in the disease’s onset and progression [11]. Mitochondria, essential for maintaining neuronal energy homeostasis, synaptic plasticity, and neurotransmitter regulation, are especially vulnerable in the AD brain due to their role in high-energy-demand processes. This vulnerability suggests that mitochondrial dysfunction, coupled with metabolic disturbances, might be among the earliest drivers of neurodegeneration in AD [76].

Neurons are highly reliant on oxidative phosphorylation for ATP production, and, in the context of AD, there is a significant reduction in mitochondrial function. Studies have shown that impaired mitochondrial dynamics—particularly the processes of mitochondrial biogenesis, fission, and fusion—are evident in the brains of individuals with AD [11]. These alterations result in decreased ATP production, which, in turn, compromises the energy required for essential neuronal processes, such as synaptic transmission and the maintenance of ionic gradients (224). This energetic failure exacerbates neuronal vulnerability, leading to progressive synaptic dysfunction, which is a key feature of the cognitive decline observed in AD [11].

One of the critical issues in AD is the increase in reactive oxygen species (ROS) production, which occurs as a byproduct of mitochondrial dysfunction. Elevated ROS levels lead to oxidative stress, which further damages cellular components, including lipids, proteins, and DNA [127,128]. This oxidative damage accelerates the progression of AD by promoting Aβ aggregation and tau hyperphosphorylation, two pathological features that are closely associated with neurodegeneration [127,128]. Oxidative stress also disrupts calcium homeostasis, which is essential for neuronal signaling and mitochondrial function. As mitochondria become increasingly dysfunctional, they are less able to regulate intracellular calcium levels, contributing to further cellular damage and apoptotic pathway activation [127,128].

Additionally, it is essential to address the significant role of lipids in the pathology and progression of this condition. Lipid dysregulation, particularly involving oxysterols such as 7-ketocholesterol, has been identified as a critical factor in both aging and neurodegenerative diseases. Anderson et al. (2020) highlight the oxidative effects of 7-ketocholesterol, which contribute to cellular stress, inflammation, and apoptosis, all of which are implicated in Alzheimer’s disease progression [129]. Similarly, there is provided evidence linking oxysterols to age-related diseases, underscoring their role in neuroinflammation and metabolic disruptions [11]. Also, it has been explored that there are lipid biomarkers specific to Alzheimer’s disease, emphasizing the potential of these molecules as diagnostic tools and therapeutic targets. Including this information enriches the discussion on Alzheimer’s disease metabolism by integrating the pivotal role of lipids and their contribution to the disease’s molecular and clinical landscape [130].

Moreover, evidence suggests that mitochondrial DNA (mtDNA) mutations and deletions are more common in individuals with AD, leading to impaired electron transport chain (ETC) function and reduced mitochondrial efficiency [11,75]. The ETC is crucial for ATP production, and, when its function is compromised, neurons experience an energy deficit that exacerbates their already compromised ability to maintain proper function in the face of accumulating AD pathology [128]. Additionally, these mtDNA mutations can lead to further increases in ROS production, perpetuating a vicious cycle of oxidative damage and mitochondrial impairment.

Recent studies also highlight the role of amyloid precursor protein (APP) and Aβ in directly impairing mitochondrial function. Aβ can localize to mitochondria, where it disrupts the ETC and inhibits key enzymes involved in oxidative phosphorylation, further reducing ATP production and increasing ROS levels (231). This mitochondrial Aβ accumulation not only contributes to cellular energy deficits but also promotes the pathological processes that characterize AD, including synaptic loss and cognitive decline [128]. Additionally, tau protein, when hyperphosphorylated, can affect mitochondrial transport along axons, leading to impaired energy distribution within neurons and contributing to the synaptic degeneration seen in AD [128].

The role of mitochondrial dysfunction in AD extends beyond energy production. Mitochondria also play a role in the regulation of apoptosis, and their dysfunction in AD can trigger apoptotic pathways, leading to neuronal death [128]. As the disease progresses, the accumulation of damaged mitochondria further compromises cellular function, leading to a cascade of neurodegeneration. Mitophagy, the process by which damaged mitochondria are degraded and removed, is also impaired in AD, leading to the accumulation of dysfunctional mitochondria that contribute to the disease’s pathology [76,128].

Interventions targeting the mitochondrial function and cellular metabolism in AD are of growing interest. Studies exploring the potential of physical activity as a non-pharmacological intervention have shown promising results in mitigating mitochondrial dysfunction [128]. Exercise promotes mitochondrial biogenesis, enhances oxidative phosphorylation, and reduces oxidative stress, which could delay or reduce the severity of AD symptoms [118,128]. By promoting healthy mitochondrial turnover and improving overall energy metabolism, exercise may help maintain neuronal function in the face of the metabolic challenges posed by AD. Furthermore, the combination of physical activity with nutritional strategies aimed at improving mitochondrial health—such as diets rich in antioxidants and anti-inflammatory compounds—may offer a more comprehensive approach to managing AD [128].

In summary, mitochondrial dysfunction and altered cellular metabolism are central to the progression of Alzheimer’s disease. These disturbances not only exacerbate the accumulation of classical AD pathologies, such as Aβ plaques and tau tangles, but also contribute to the energy deficits, oxidative stress, and neuronal death that underlie cognitive decline. Addressing these metabolic issues through lifestyle interventions, including physical activity and dietary modifications, could offer promising avenues for slowing the progression of AD and improving the quality of life for affected individuals. Future research should focus on optimizing these interventions and exploring the potential of biomarkers related to mitochondrial function to provide personalized therapeutic approaches [14,128].

## 7. Cell Metabolism in Parkinson’s Disease

Parkinson’s disease (PD) is a progressive neurodegenerative disorder that primarily affects movement, leading to symptoms such as bradykinesia, tremors, rigidity, and postural instability. These motor symptoms are caused by the loss of dopaminergic neurons in the substantia nigra pars compacta, a brain region responsible for controlling movement. However, beyond these hallmark motor features, PD is also marked by significant cellular metabolic dysfunctions that contribute to non-motor symptoms, including cognitive decline, depression, sleep disturbances, and autonomic dysfunctions [131]. Understanding the cellular metabolism in PD, particularly mitochondrial dysfunction and oxidative stress, is essential for advancing therapeutic strategies aimed at halting or slowing down the progression of the disease.

The link between mitochondrial dysfunction and PD was first identified in the 1980s, when post-mortem studies revealed the impaired activity of complex I in the electron transport chain (ETC) of the substantia nigra in patients with PD [132]. Complex I is the first enzyme in the mitochondrial respiratory chain, and its dysfunction severely impairs ATP production. This defect is critical because neurons, especially dopaminergic neurons, rely heavily on mitochondrial oxidative phosphorylation to generate energy. These neurons have exceptionally high metabolic demands due to their extensive synaptic activity and long axonal projections. In PD, the impairment of complex I reduces ATP production and increases the production of reactive oxygen species (ROS), leading to oxidative stress and neuronal damage [133].

Oxidative stress, caused by an imbalance between ROS production and the cell’s ability to neutralize these reactive molecules, plays a pivotal role in the degeneration of dopaminergic neurons in PD. Excessive ROS can damage cellular structures, including lipids, proteins, and DNA, further exacerbating mitochondrial dysfunction and neuronal death. Dopaminergic neurons are particularly vulnerable to oxidative stress due to the metabolism of dopamine itself, which can generate ROS as a byproduct. Additionally, the accumulation of misfolded alpha-synuclein, a hallmark protein in PD pathology, also disrupts mitochondrial function by promoting oxidative damage and impairing mitochondrial dynamics [134].

Alpha-synuclein aggregates, which form Lewy bodies in the brains of PD patients, are known to interact with mitochondria and further compromise their function. Studies have shown that alpha-synuclein can localize to the inner mitochondrial membrane, where it disrupts the electron transport chain, particularly at complex I, further reducing ATP production and increasing oxidative stress [133]. This interaction between alpha-synuclein and mitochondria is thought to create a vicious cycle in which mitochondrial dysfunction promotes alpha-synuclein aggregation, and alpha-synuclein, in turn, exacerbates mitochondrial dysfunction. This cycle is central to the progressive nature of PD, as mitochondrial damage accumulates over time, leading to the loss of dopaminergic neurons. Moreover, mitochondrial DNA (mtDNA) mutations and deletions have been observed in the substantia nigra of PD patients. These mutations can impair mitochondrial function, reducing the efficiency of oxidative phosphorylation and increasing ROS production. Studies have shown that mtDNA mutations accumulate with age, and their presence is correlated with the increased vulnerability of neurons to oxidative stress and metabolic dysfunction. Given that aging is one of the most significant risk factors for PD, these findings suggest that mitochondrial DNA damage is a key contributor to the development and progression of the disease [135].

In addition to mitochondrial dysfunction, impaired mitophagy—the process by which damaged mitochondria are degraded and removed—has also been implicated in PD. Mitophagy is essential for maintaining mitochondrial quality control and ensuring that damaged or dysfunctional mitochondria do not accumulate within cells. In PD, mutations in genes such as PINK1 (PTEN-induced putative kinase 1) and Parkin, which are key regulators of mitophagy, lead to the defective clearance of damaged mitochondria. This failure in mitophagy allows dysfunctional mitochondria to persist, further increasing oxidative stress and contributing to neuronal death (245). PINK1 and Parkin mutations are associated with familial forms of PD, but defects in mitophagy have also been observed in sporadic cases, underscoring the importance of mitochondrial quality control in the broader context of PD pathogenesis.

Another critical aspect of cellular metabolism in PD is the role of calcium homeostasis. Mitochondria play a central role in buffering intracellular calcium levels, which is essential for maintaining neuronal excitability and synaptic function. In PD, mitochondrial dysfunction impairs calcium handling, leading to elevated intracellular calcium levels that can trigger apoptotic cell death pathways. Dopaminergic neurons in the substantia nigra are particularly susceptible to disruptions in calcium homeostasis due to their reliance on calcium-dependent pacemaking activity for dopamine release (246). As mitochondrial calcium buffering capacity declines in PD, neurons become more vulnerable to excitotoxicity, further contributing to their degeneration. The impairment of energy metabolism in PD is not limited to the substantia nigra but also affects other brain regions, contributing to the non-motor symptoms of the disease. For example, mitochondrial dysfunction has been observed in the hippocampus and cerebral cortex, which are involved in memory and cognition. This finding suggests that metabolic disturbances in these regions may contribute to the cognitive decline observed in PD patients (247). Additionally, energy deficits in peripheral tissues, such as muscle, may underlie the fatigue and muscle weakness often experienced by individuals with PD.

Therapeutic strategies targeting mitochondrial dysfunction and cellular metabolism are of growing interest in the treatment of PD. Interventions aimed at enhancing mitochondrial function, reducing oxidative stress, and improving mitophagy have shown promise in preclinical models of PD. For example, compounds that boost the activity of PGC-1α, a transcriptional coactivator that regulates mitochondrial biogenesis, have been shown to protect dopaminergic neurons by promoting the generation of new, healthy mitochondria [136]. Similarly, antioxidants that neutralize ROS have been explored as potential therapies for reducing oxidative damage in PD, although clinical trials have yielded mixed results [137].

Physical activity is emerging as a potent non-pharmacological intervention for mitigating mitochondrial dysfunction in PD. Exercise has been shown to promote mitochondrial biogenesis, enhance oxidative phosphorylation, and reduce oxidative stress, which may help preserve dopaminergic neurons and slow disease progression. Regular physical activity has also been associated with improved motor function, reduced fatigue, and better overall quality of life in PD patients. Additionally, combining physical activity with nutritional interventions aimed at supporting mitochondrial health, such as diets rich in antioxidants and anti-inflammatory compounds, may offer a comprehensive approach to managing PD [116].

Thus, mitochondrial dysfunction, impaired energy metabolism, and oxidative stress are central to the pathogenesis of Parkinson’s disease. These metabolic disturbances not only contribute to the motor symptoms of PD but also underlie many of the non-motor symptoms that affect patients’ quality of life. Addressing these metabolic issues through therapeutic interventions that target mitochondrial function, enhance mitophagy, and reduce oxidative stress holds promise for slowing the progression of PD and improving patient outcomes. Future research should continue to explore the potential of combining lifestyle interventions, such as exercise and dietary modifications, with pharmacological approaches to provide a more holistic strategy for managing Parkinson’s disease [138].

## 8. Cell Metabolism in Multiple Sclerosis

Multiple Sclerosis is a complex, chronic neurological disorder characterized by an interplay of immune-mediated inflammation, demyelination, and progressive neurodegeneration. This multifactorial disease affects millions of people worldwide and is marked by significant variability in its clinical presentation, disease course, and response to treatment [13]. The progressive nature of Multiple Sclerosis and its impact on the CNS have incited extensive research into the underlying mechanisms that drive the disease. Among these, a growing body of evidence implicates mitochondrial dysfunction as a central element in the pathophysiology of Multiple Sclerosis, highlighting the critical role of energy metabolism in the disease’s progression. Mitochondria, the organelles responsible for cellular energy production through oxidative phosphorylation, are particularly vulnerable in this disease due to the heightened metabolic demands imposed by the disease’s pathological processes [139]. In affected individuals, mitochondrial function is significantly compromised, as evidenced by impaired oxidative phosphorylation. The reduction in ATP production in neurons and glial cells, coupled with the increased production of ROS, creates a metabolic environment inclined to cellular stress and damage. These energy deficits are especially detrimental in the context of demyelination, where axons, stripped of their myelin sheath, become heavily reliant on mitochondria for maintaining energy homeostasis [139]. This increased energy demand, in the face of impaired mitochondrial function, exacerbates axonal degeneration, a key pathological feature of Multiple Sclerosis. This degeneration is not merely a consequence of inflammatory processes but is also driven by metabolic disturbances, placing mitochondrial dysfunction as the main feature in Multiple Sclerosis pathology [83].

The significance of mitochondrial dysfunction in this disease is further underscored by its impact on a range of CNS cell types, including neurons, oligodendrocytes, and astrocytes, all of which are heavily reliant on functional mitochondria for their energy needs. Neurons, particularly those in demyelinated regions, require substantial ATP to maintain ionic gradients necessary for action potential propagation [140,141]. Oligodendrocytes, responsible for myelination, also depend on mitochondrial function to support the energy demanding process of myelin repair and maintenance. Astrocytes, which play a critical role in maintaining the CNS environment, also rely on mitochondrial activity to support their various functions, including the regulation of neurotransmitter levels and the maintenance of the blood–brain barrier [142]. The cumulative effect of impaired mitochondrial function across these cell types leads to a widespread energy deficit in the brain and spinal cord, contributing to the broad spectrum of neurological symptoms observed in Multiple Sclerosis. Additionally, the chronic production of ROS by dysfunctional mitochondria exacerbates oxidative stress, which further damages cellular structures, including lipids, proteins, and DNA. This oxidative damage not only impairs cell function but also triggers apoptotic pathways, leading to cell death. The combination of energy deficits, oxidative stress, and subsequent cell loss creates a vicious cycle that propels the neurodegenerative processes, making the understanding and targeting of mitochondrial dysfunction critical for therapeutic development.

The role of mitochondrial dysfunction in Multiple Sclerosis is further complicated by its interactions with other pathological processes, such as neuroinflammation and excitotoxicity. Neuroinflammation, a hallmark of this disease, is characterized by the infiltration of immune cells into the CNS and the activation of resident microglia and astrocytes. This inflammatory response, while initially protective, can become chronic and deleterious, exacerbating mitochondrial damage and contributing to the disease’s progression [143]. For example, pro-inflammatory cytokines released during neuroinflammation can impair mitochondrial function by disrupting electron transport chain activity, increasing ROS production, and promoting the release of pro-apoptotic factors from mitochondria. These changes not only contribute to the direct damage of CNS cells but also amplify the inflammatory response, creating a feedback loop that perpetuates neurodegeneration [144]. Excitotoxicity, another process implicated, involves the excessive activation of glutamate receptors, leading to increased intracellular calcium levels and further mitochondrial dysfunction. The convergence of these processes highlights the central role of mitochondria in the pathophysiology of Multiple Sclerosis and underscores the importance of developing therapies that target mitochondrial health [145].

Physical activity has emerged as a promising therapeutic intervention, largely due to its ability to modulate key metabolic pathways and improve mitochondrial function. Exercise exerts a protective effect on mitochondria by enhancing mitochondrial biogenesis, the process by which new mitochondria are formed within cells. This process is driven by the upregulation of PGC-1α. Increased PGC-1α expression in response to physical activity promotes the replication and growth of mitochondria, thereby improving the overall capacity of cells to produce ATP [146]. This enhancement in mitochondrial function is particularly beneficial for Multiple Sclerosis patients, as it helps to counteract the energy deficits caused by impaired oxidative phosphorylation. Moreover, exercise facilitates the removal of damaged mitochondria through a process known as mitophagy, thereby reducing the accumulation of dysfunctional mitochondria that could contribute to cellular stress and degeneration. By promoting the turnover of mitochondria, exercise not only supports energy production but also mitigates the oxidative stress associated with mitochondrial dysfunction. This dual benefit of exercise enhancing mitochondrial function while reducing oxidative damage provides a powerful strategy for addressing some of the core metabolic challenges in Multiple Sclerosis. The benefits of exercise extend beyond its effects on mitochondrial biogenesis and function. Clinical studies have shown that regular physical activity can lead to significant improvements in motor function, reduction in fatigue, and enhanced quality of life for individuals with Multiple Sclerosis [42]. These improvements are thought to be mediated by the neuroprotective effects of exercise, which include not only enhanced mitochondrial function but also improved synaptic plasticity, reduced neuroinflammation, and better myelin repair. The upregulation of PGC-1α and other exercise-induced signaling pathways contributes to these beneficial outcomes by supporting neuronal survival and function in the face of the metabolic challenges posed by Multiple Sclerosis. Furthermore, the ability of exercise to reduce oxidative stress through the upregulation of antioxidant defenses provides an additional layer of protection against the cellular damage that drives Multiple Sclerosis progression [147]. Collectively, these findings underscore the potential of exercise as a therapeutic strategy that targets the underlying metabolic disturbances in Multiple Sclerosis, offering a non-pharmacological approach to managing the disease.

In addition to mitochondrial dysfunction, alterations in glycolytic pathways have also been observed in Multiple Sclerosis, further contributing to the complex metabolic landscape of the disease. Anaerobic glycolysis is typically upregulated in immune cells and neurons under conditions of stress or high energy demand [148]. In Multiple Sclerosis, there is a noticeable shift from aerobic to anaerobic metabolism, particularly in immune cells such as macrophages and microglia, as well as in neurons. This metabolic reprogramming is thought to support the heightened inflammatory response observed in Multiple Sclerosis, as it provides the rapid energy needed for immune cell activation and proliferation. However, this shift also contributes to the inflammatory and neurodegenerative processes in Multiple Sclerosis by promoting a pro-inflammatory environment and reducing the efficiency of ATP production [149]. Exercise has been shown to have a normalizing effect on these metabolic shifts, helping to restore a more balanced energy metabolism in both immune and neural cells. This metabolic rebalancing is crucial for reducing inflammation and protecting neurons from degeneration. Additionally, the enhanced mitochondrial function induced by exercise may further support this shift by improving the efficiency of ATP production and reducing the production of ROS, thereby mitigating some of the key metabolic disturbances that drive Multiple Sclerosis pathology. Through these mechanisms, exercise not only improves overall metabolic health but also offers a targeted approach to addressing the specific metabolic challenges associated with Multiple Sclerosis [150].

Recent advancements in understanding the metabolic foundations of Multiple Sclerosis have also led to the exploration of potential biomarkers that could aid in the diagnosis and monitoring of the disease. Metabolic biomarkers, such as lactate levels, mitochondrial DNA mutations, and specific lipid profiles, are being investigated for their ability to reflect the metabolic state of CNS cells [151]. These biomarkers could provide valuable insights into disease progression and the effectiveness of therapeutic interventions, including exercise. Additionally, novel imaging techniques, such as magnetic resonance spectroscopy (MRS), are being developed to assess metabolic changes in the brain and spinal cord of Multiple Sclerosis patients [152]. These techniques offer the potential to non-invasively monitor mitochondrial function and other metabolic processes, providing a more comprehensive understanding of the disease and guiding personalized treatment strategies.

The relationship between cell metabolism and Multiple Sclerosis is deeply intertwined, with mitochondrial dysfunction, altered metabolism, and oxidative stress playing central roles in the disease’s pathophysiology. The therapeutic potential of exercise lies in its ability to modulate these metabolic pathways, offering a multifaceted approach to disease management. By improving mitochondrial function, enhancing energy production, and reducing oxidative stress, physical activity can help to counteract the metabolic disturbances that contribute to neurodegeneration in Multiple Sclerosis. As our understanding of these metabolic processes continues to evolve, so too does the potential for developing more effective therapeutic strategies. The integration of metabolic therapies with existing treatments, along with the development of new diagnostic tools, holds promise for improving outcomes and quality of life for individuals living with this disease.

## 9. Specific Metabolic Pathways Affected by Exercise

**Figure 4:**
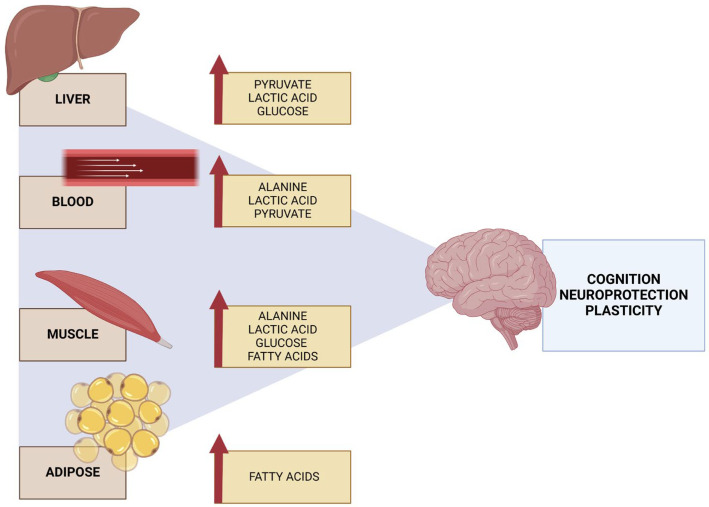
Systemic metabolic adaptations to exercise across key organs.

Exercise enhances all aspects of the human body and is crucial in the prevention of chronic diseases. More concretely, exercise profoundly impacts several metabolic pathways in the brain, enhancing energy production, promoting mitochondrial biogenesis, and reducing oxidative stress. Thyfault et al. specified that the metabolic effects of exercise on key organs are focused on the liver, which increases the production of lactic acid, pyruvate, and glucose to supply energy for active muscles, especially during prolonged activity; muscles consume more lactic acid, pyruvate, and glucose and increase fatty acid oxidation, allowing them to sustain energy by using fat as fuel, which is vital for endurance [153]. Adipose tissue releases fatty acids into the blood, providing an alternative energy source for muscles and aiding in fat loss and improved body composition; the brain responds to exercise by improving cognition [154] and neuroprotection, enhancing focus, reducing risks of neurodegenerative diseases [155], and supporting learning and adaptation. And each organ’s response works together to improve energy utilization, physical endurance, and mental resilience (Figure 4) [153].

Key pathways include AMPK activation, glycolysis, and the lactate–BDNF axis, which support neuronal health and cognitive function [156]. Additionally, exercise boosts antioxidant defenses and fatty acid oxidation while modulating the kynurenine pathway to reduce neuroinflammation [157]. These mechanisms collectively contribute to improved brain function and resilience against neurodegenerative diseases.

The effects of exercise are facilitated by a complex process that activates integrated bodily systems at the molecular and cellular levels. In this regard, the ongoing provision of adenosine triphosphate (ATP), a molecule that serves the essential cellular functions that facilitate skeletal muscle contraction during exercise, is crucial for performance in events ranging from seconds to several hours [158]. Due to the limited storage of ATP in muscle tissue, metabolic pathways must be initiated to sustain the necessary rates of ATP resynthesis. These pathways encompass the breakdown of phosphocreatine and muscle glycogen, facilitating substrate-level phosphorylation (‘anaerobic’) and oxidative phosphorylation through the utilization of reducing equivalents derived from carbohydrate and fat metabolism (‘aerobic’). The intensity and duration of physical activity chiefly influence the proportional impact of these metabolic pathways [26]. Then, well-developed control systems guarantee the rapid provision of ATP and the preservation of the ATP content in muscle cells, as the metabolic rate can increase from rest to exercise [159].

### 9.1. Intense Short-Term Exercise

When intense short-term exercise begins, both anaerobic and aerobic ATP production pathways are activated [160]. The anaerobic pathways, particularly phosphocreatine (PCr) hydrolysis and anaerobic glycolysis, provide ATP much more rapidly than aerobic processes. PCr is a remarkable fuel source, as it requires only one metabolic reaction to produce ATP, catalyzed by the enzyme creatine phosphokinase. This reaction is highly efficient, with ATP regeneration occurring in just a few milliseconds [161].

As muscle contractions begin, the concentration of free (Adenosine di-phosphate) ADP increases, driving the reaction from left to right, and ATP is quickly regenerated. Simultaneously, cellular Ca2+ and epinephrine activate phosphorylase kinase, converting glycogen phosphorylase from its less active ‘b’ form to its more active ‘a’ form. This activation, along with increases in ADP and AMP, enhances the breakdown of glycogen, leading to the production of glucose 1-phosphate, glucose 6-phosphate, and fructose 6-phosphate in the glycolytic pathway [162,163].

The regulatory enzyme phosphofructokinase is activated by the allosteric regulators ADP, AMP, and Pi, as well as the substrate fructose 6-phosphate. This activation drives the glycolytic pathway, resulting in the net production of three ATP molecules and lactate formation. Despite the multiple reactions involved, anaerobic glycolysis can also produce ATP within milliseconds [163].

Lactate accumulation can be detected in the muscle after just a 1 s contraction, and the contributions of anaerobic energy from PCr and anaerobic glycolysis are essentially equivalent after 6–10 s of intense exercise [164]. The capacity of the PCr energy storage, which is a function of its resting content, can be mostly depleted in 10–15 s of all-out exercise. The anaerobic glycolytic capacity, which is approximately threefold higher, is limited not by glycogen availability but by increasing intramuscular acidity [26,165].

Aerobic ATP production is also activated during very intense exercise, with 70–100% of VO2 max reached in an all-out 30 s sprint [166]. Although very little aerobic energy is provided in the first 5–10 s, about 50% of the energy contribution in the last 5 s of a 30 s sprint is aerobic [165]. During the transition from rest to intense exercise, the substrate for increased aerobic ATP production is muscle glycogen. A small amount of the produced pyruvate is transferred into the mitochondria, where it is used to produce acetyl-CoA and the reducing equivalent NADH in the pyruvate dehydrogenase (PDH) reaction. PDH is also under covalent control, existing in an inactive form at rest and transitioning to an active form via Ca2+ during exercise. The influence of Ca2+, with help from pyruvate, keeps the appropriate amount of the enzyme in the active form, despite increases in acetyl-CoA that would normally inactivate the enzyme at rest [167].

It has been concluded that intense short-term exercise activates both anaerobic and aerobic ATP production pathways, with rapid energy supplied by phosphocreatine hydrolysis and glycolysis. The resulting metabolic processes, including lactate production and enhanced mitochondrial activity, not only meet the immediate energy demands but also contribute to improved cellular function [168]. This dynamic shift in energy metabolism highlights the integral role of exercise in supporting both muscle performance and broader metabolic adaptations, particularly in brain health and disease prevention.

### 9.2. Aerobic Exercise

During endurance exercise below 100% VO2 max, aerobic ATP generation is dominant. In this scenario, there is sufficient time to mobilize fat and carbohydrate substrates from muscle, adipose tissue, and the liver. Initially, muscles rely on anaerobic energy for the first 1–2 min when transitioning from rest to aerobic power output, but then aerobic metabolism takes over. The respiratory or electron transport chain in the mitochondria requires NADH, FADH2, ADP, Pi, and O2 to produce ATP. The respiratory and cardiovascular systems ensure O2 delivery to contracting muscles, and the by-products of ATP utilization (ADP and Pi) are transported back into the mitochondria for ATP resynthesis [169]. The processes that move ATP out of the mitochondria, ADP, and Pi back in are highly regulated [170,171].

The tricarboxylic acid (TCA) cycle in the mitochondria produces reducing equivalents and accepts acetyl-CoA mainly from carbohydrates and fats. During aerobic exercise, increased mitochondrial Ca2+ concentrations activate TCA cycle enzymes [172]. Substrate accumulation and local regulators fine-tune the flux through the dehydrogenases, and citrate synthase controls the TCA cycle flux. Ca2+ also activates glycogen phosphorylase and PDH [173], but the glycolytic flux needed to supply acetyl-CoA from carbohydrates and, ultimately, aerobic ATP production is lower than during sprint exercise [26].

Ca2+ and epinephrine activate enzymes regulating IMTG degradation, and fatty acids are provided in the cytoplasm [174]. Ca2+ contributes to the movement of glucose and fatty acids into muscle cells. The transport protein GLUT4 facilitates glucose influx, and increased glucose delivery and metabolism maintain the gradient for glucose diffusion during exercise [30]. The translocation of GLUT4 is a key event in exercise-induced muscle glucose uptake [175].

Fatty acids are transported into cells and mitochondria by transport proteins for fat, which are translocated to the muscle and mitochondrial membranes [176]. The CPT I system and fat-transport proteins are also required to facilitate the transportation of fatty acids across the mitochondrial membranes. Upon entering the mitochondria, fat undergoes the β-oxidation pathway, which results in the production of acetyl-CoA and reducing equivalents, thereby generating substantial quantities of aerobic ATP [177]. Substrates are contributed by both fat and carbohydrate at moderate exercise intensities (~50–70% VO2 max). Nevertheless, during high-intensity endurance events, the body’s reliance on fat decreases, and fuel consumption moves toward carbohydrates. From a performance standpoint, this fuel shift is logical, as carbohydrate aerobic ATP production is more efficient than fat. Nevertheless, athletes may be compelled to reduce their pace if the event is prolonged, as glycogen stores may be depleted. Research has identified numerous sites where fat metabolism is downregulated at high aerobic exercise intensities, such as the inhibition of CPT I activity, decreased fatty acid release from adipose tissue, and reduced IMTG breakdown [178]. As aerobic metabolism dominates during endurance exercise, the enhanced mitochondrial activity and efficient ATP production contribute to the regulation of inflammatory responses. This process, combined with improved fatty acid oxidation and glucose metabolism, helps reduce neuroinflammation by lowering pro-inflammatory cytokines and oxidative stress, ultimately supporting brain health [168].

## 10. Neuroprotective Benefits of Exercise

Consistent physical activity is linked to significant health advantages [179]. The molecular mechanisms underlying physiological adaptations to exercise are most comprehensively elucidated in skeletal muscle [180]. Augmented mitochondrial functions in muscle are pivotal to exercise-induced adaptations. Regular exercise also enhances cognitive function and serves as a significant protective factor against neurodegenerative diseases, including the prevalent age-related dementia, Alzheimer’s disease, and the most common neurodegenerative motor disorder, Parkinson’s disease [14,180].

### 10.1. Exercise as a Protector

Despite the significant differences in pathology and symptomatology among various neurodegenerative diseases, several noteworthy commonalities are present. Concretely, mitochondrial dysfunction, encompassing deficiencies in mitochondrial metabolism, respiration, dynamics, redox regulation, ion homeostasis, and cell death regulation, is central to the etiology of most neurodegenerative diseases [181,182]. For instance, mitochondrial abnormalities have been documented early in the substantia nigra of Parkinson’s disease and in the cortex of Alzheimer’s disease and Huntington’s disease patients [14].

The advantages of exercise on mitochondria are most clearly observed in skeletal muscle. Muscle mitochondria govern skeletal muscle mass and function and are subsequently influenced by exercise [183]. Exercise promotes mitochondrial plasticity and enhances mitochondrial biogenesis and respiration [24,184]. It also augments antioxidant capabilities and mitochondrial affinity for oxygen, thereby enhancing fatty acid oxidation, aerobic performance, overall health, and promoting healthy aging [185,186,187]. These mechanisms fundamentally rely on mitochondrial integrity and quality control, as well as the mitochondria’s ability to appropriately alter their morphology, augment their quantity, and improve their mobility and distribution within cells. Specifically, studies conducted by Bishop and colleagues, as well as Hood and associates, have evidenced that a singular session of high-intensity exercise suffices to enhance the expression of proteins associated with mitochondrial biogenesis and energy production, stimulate the translation of oxidative phosphorylation-related proteins, and augment ATP synthesis in skeletal muscle [188,189]. Regarding neurobenefits, mitochondrial plasticity in response to exercise is primarily recognized in skeletal muscle, though it has also been documented in other tissues, such as the brain [190,191,192,193]. Comparable to the effects observed in skeletal muscle, exercise-induced augmentation of the mitochondrial electron transport system, biogenesis, and antioxidative capacities has been documented in the murine brain. Transient elevations in reactive oxygen species (ROS) levels induced by exercise can influence redox regulation, including in the brain, similar to skeletal muscle [194,195], and can also provoke adaptive responses that enhance endogenous antioxidant capacities [196]. Regular exercise likely safeguards against neurodegenerative diseases through both the enhancement of antioxidative stress defenses and associated advantageous mitochondrial adaptations, as well as indirectly, by modulating neuroprotective factors, such as the brain-derived neurotrophic factor (BDNF) via reactive oxygen species (ROS) [180,197].

Additionally, mitochondria are increasingly recognized for their role in regulating numerous intracellular processes and for their capacity to influence both mitochondria and cells in remote locations, thereby playing significant communicative roles across cellular boundaries. They seem to be synchronized across cells without direct contact, a phenomenon that remains poorly understood [198,199]. Thus, mitochondria not only release and respond to signaling molecules, but they can also be transferred between cells as entire organelles or organelle components. The transfer of mitochondria among various cell types encompasses, for instance, the exchange between astrocytes and neurons to facilitate the degradation of impaired neuronal mitochondria or to provide support to neurons with astrocytic mitochondria, such as following a stroke [14,200,201]. Moreover, the administration of exogenous isolated mitochondria or mitochondrial transfer from external stem cells has yielded advantageous results for brain tissues [202,203].

### Exerkines

The health advantages of exercise are widely acknowledged and evident across various organ systems. These advantageous effects augment overall resilience, healthspan, and longevity. The molecular mechanisms that elucidate the advantageous effects of exercise, however, remain inadequately comprehended. Following the 2000 discovery that muscle contraction releases IL-6, the identification of exercise-associated signaling molecules has significantly increased. Exerkines are signaling molecules released in response to acute and/or chronic exercise, which exert their effects via endocrine, paracrine, and/or autocrine pathways. A variety of organs, cells, and tissues secrete these factors, including skeletal muscle (myokines), the heart (cardiokines), liver (hepatokines), white adipose tissue (adipokines), brown adipose tissue (baptokines), and neurons (neurokines). Thus, exerkines possess potential functions in enhancing cardiovascular, metabolic, immune, and neurological health [204].

In this regard, contracting muscle fibers generate and secrete myokines that are crucial for skeletal muscle communication with other organs and tissues. The functional outcomes of their release are influenced by variables including exercise volume, intensity, and frequency [205]. A variety of these exercise-induced myokines have been identified, including irisin, cathepsin B, fibroblast growth factor 21 (FGF-21), and the BDNF, collectively referred to as the “myokinome” [206]. All engage in systemic exercise signaling, with certain individuals specifically associated with central nervous system effects through the modulation of adult neurogenesis and cognitive function. This finding indicates that these myokines partially facilitate the cognitive advantages associated with regular exercise [207,208].

The initial identified and extensively researched myokine is interleukin-6 (IL-6). The function of IL-6 in skeletal muscle adaptations to exercise has been thoroughly established. Skeletal muscle contraction induces IL-6 independently of tumor necrosis factor-α [209], indicating that muscle-derived IL-6 enhances the metabolic function rather than the inflammatory function. Numerous studies have shown that peripherally secreted IL-6 traverses the blood–brain barrier and modulates food consumption, potentially mitigating obesity and obesity-related neurological disorders [210]. Brooks and colleagues demonstrated that lactate is a principal exerkine produced by skeletal muscle in response to exercise [211]. In this regard, evidence is mounting that lactate derived from exercise traverses the blood–brain barrier and activates BDNF-mediated signaling in the hippocampus, enhancing learning and memory. Moreover, lactate also directly enhances metabolic activity in brain regions, including the hypothalamus and hippocampus. The hypothalamus detects lactate in the bloodstream, regulating the glucose production essential for glucose homeostasis [212].

Additionally, Cheng and colleagues reported that the BDNF activates peroxisome proliferator-activated receptor gamma coactivator 1 alpha (PGC1α), leading to enhanced mitochondrial biogenesis and a concomitant rise in cellular energy substrates, including ATP and NAD+, alongside the preservation and formation of synapses [209,213]. Rody et al. also emphasized recently the neuroprotective function of irisin, a myokine derived from the proteolytic cleavage of the fibronectin type III domain-containing protein 5 (FNDC5) transmembrane protein, showing the evidence that physical exercise may serve as a preventive and therapeutic approach to cognitive decline in Alzheimer’s disease [214]. Although Irisin/BDNF signaling appears to be a significant mediator of communication between contracted skeletal muscles and the brain during exercise training [215], and there are well-defined outcomes, such as those related to hippocampal plasticity and memory [216], the mechanistic comprehension of myokine-related effects on the brain remains inadequate [217]. This finding encompasses molecular modifications, including diminished ROS production and oxidative injury, alongside enhanced enzymatic antioxidant defenses, significant impacts on elements associated with mitochondrial biogenesis, and effects on hippocampal adult neurogenesis [218].

In conclusion, the function of mitochondria and mitochondrial stress in skeletal muscle regarding exercise’s impact on brain function remains relevant. Thus, mitochondria play a crucial role in the skeletal muscle’s response to exercise; however, the mechanisms by which they engage in systemic signaling, especially to the brain, are yet to be clarified. The roles of myokines and mitochondrial transfer in exercise signaling, particularly concerning the brain, represent compelling subjects for future investigation. Nonetheless, it is evident that the potentially neuroprotective effects of exercise-induced signals to the brain contribute to the enhanced or sustained brain function, including improved cognitive abilities linked to the hippocampus and the increased resilience of hypothalamic neurons responsible for the hormonal regulation of hunger and satiety against the adverse effects of a high-fat diet [219]. Mitochondrial reprogramming, improved waste clearance, and enhanced nutrient and oxygen supply due to exercise may augment mitochondrial function, serving as a potential mechanism for the cognitive benefits of exercise [220].

## 11. Therapeutic Interventions Based on Exercise

Scientific research has extensively demonstrated the critical role of physical exercise in promoting cardiovascular health. Going forward, we will focus on the recent advances that underscore the additional benefits of this habit for mental health, acquired brain injury, and neurodegeneration [221]. But what makes exercise such a powerful tool? The answer is multifaceted and presents significant challenges for modern science.

It has been shown that exercise stimulates the release of neurotrophic factors linked to neuronal plasticity and neurogenesis [207]. Additionally, it is associated with increased serotonin levels, thereby improving mood by reducing anxiety, depression, and stress [6,7]. Muscle contraction also triggers the secretion and release of myokines, which are beneficial for the brain and are closely connected to muscle–brain communication [222]. This communication may also be key in the interaction between muscle and adipose tissue, reducing adipokines involved in harmful inflammatory processes affecting the brain [223].

In this context, exosomes have been proposed as fundamental components in the communication between tissues and muscles during physical exercise [17], highlighting their potential role in the release of myokines triggered by exercise. One notable myokine is irisin, encoded by the *FNDC5* gene, whose benefits are explained by the positive correlation between its circulating levels, skeletal muscle, and aerobic capacity. It may also enhance synaptic capacity and memory in Alzheimer’s disease (AD) [118]. In fact, impaired neuroplasticity and the accumulation of various adverse factors are characteristic of AD. Furthermore, several metabolic disorders are associated with AD and brain health, including those related to lipid metabolism, glucose metabolism, amyloid-beta (Aβ) transport, and iron and tau protein metabolism.

While physical exercise can markedly improve these deteriorations, current science is still unraveling the mysteries behind these profound benefits. In this context, various metabolites play a critical role. Lactate, a paradigm within the framework of physical exercise, may be responsible for activating the brain-derived neurotrophic factor (BDNF) or abrineurin expression during physical activity, thereby enhancing learning capacity and memory skills [224]. There is also a protease called cathepsin B, which is significant because it increases with exercise and stimulates neurogenesis. Cathepsin B also influences processes related to antigens involved in the immune response, bone turnover, hormone activation, and intracellular protein catabolism [225].

Another metabolite implicated in the immune response and central nervous system is kynurenine. Notably, kynurenine aminotransferases increase in skeletal muscle that is regularly trained through physical exercise [226]. Thus, the therapeutic potential of physical exercise is broad. In this regard, iron plays a key role in muscle–brain function during exercise. It can cross the blood–brain barrier and contribute to processes like respiratory chain transmission, axonal myelination, and neurotransmitter generation in the brain. Therefore, therapeutic interventions involving iron and physical exercise could have beneficial effects on patients suffering from mood disorders and neurodegenerative diseases [227].

The pathophysiology of neurodegeneration is complex and under continuous review. Mitochondrial dysfunction significantly contributes to neurodegenerative development [228]. Physical exercise provides an opportunity to positively affect mitochondrial function through metabolic regulation, promoting mitochondrial fission, biogenesis, and oxidative phosphorylation metabolism. It enhances adult hippocampal neurogenesis under both physiological and pathological conditions [187].

In fact, the benefits of exercise extend beyond purely pathophysiological processes. Intense aerobic exercise is particularly recommended for acquired brain injuries, improving fatigue levels and cognitive function in patients with moderate to severe traumatic brain injuries [229]. Additionally, exercise therapies, such as high-intensity interval training (HIIT) and steady-state cardio, are being investigated as potential interventions for neuropathic pain. In individuals with Multiple Sclerosis, acute exercise can reduce plasma neurofilament light chain levels while enhancing the kynurenine pathway flow [230]. Also, it shows potential for enhancing neuroplasticity in human Parkinson’s disease, with minimal training duration and decreased burden [231].

However, as we have outlined, several mechanisms explain why physical exercise is so beneficial for brain health. When considering therapeutic interventions, the characteristics of the exercise itself—such as type, cadence, intensity, and volume—must be evaluated to determine the most appropriate approach for each individual case.

From a therapeutic standpoint, it is crucial to assess whether exercise can serve as a concomitant or even substitute treatment, as demonstrated in mood disorders where exercise has proven to be an effective non-pharmacological strategy for rehabilitation in substance use disorder [232]. Additionally, exercise can extend brain health against the undesirable effects of aging. However, the benefits are not uniform, with variables such as sex being non-modifiable, while others present opportunities for targeted interventions. Specifically, the review and meta-analysis by Barha et al. (2017) suggest that combining different types of exercise yields greater benefits across various brain functions in the aging process [233]. Developing biomarkers that reflect the specific capacities of each type of exercise for individual patients may open new therapeutic avenues, both preventive and treatment-oriented, in neurodegenerative processes.

## 12. The Role of Nutrition

The epidemiology of modern civilization highlights sedentarism and diet as primary culprits behind the rise in obesity, type II diabetes, and metabolic syndrome (MetS) [234]. These conditions are closely linked to severe impairments in brain function. Regarding diet, the frequent consumption of high-calorie but nutritionally poor foods provides a strong foundation for the development of these metabolic disorders, which, in turn, are connected to neurodegenerative, psychiatric, and acquired brain injury-related pathologies. The root of this connection lies in the inefficacy of molecular mechanisms regulating energy metabolism and synaptic plasticity. It is crucial to acknowledge that human brain evolution has been significantly influenced by nutrition, meaning dietary factors play a critical role in brain health and can be redirected to preserve it [235].

Nevertheless, a large portion of the global population suffers from metabolic diseases, consequently increasing their risk of developing neurodegenerative disorders, affecting both the central and peripheral nervous systems. This situation demands urgent preventive measures from public health authorities to address the epidemic [236]. The literature has long warned of the dangers posed by metabolic disorders. Specifically, MetS has been extensively studied due to its high prevalence in public health statistics over recent decades and its status as a risk factor for neurological diseases, including Alzheimer’s disease (AD), depression, and stroke. Research indicates that endothelial cell dysfunction, essential fatty acid metabolism impairments, lipid mediator defects, and ineffective insulin/leptin signaling act as catalysts for these neurological conditions [237].

Scientific advances have demonstrated that multiple interactions can influence both the genesis and development of neurological deterioration. Nutritional status and diet are key factors in this context. For instance, inadequate nutrition directly impacts insulin resistance, a known risk factor for brain health [238]. Additionally, there is a positive correlation between low BMI, malnutrition, and higher rates of dementia and mortality, as malnutrition disrupts the gut–microbiota–brain axis, accelerating neurodegeneration. On the other hand, nutrition is also studied for its protective role. The Mediterranean diet and calorie-restricted diets have been shown to prevent cognitive decline, Parkinson’s disease (PD), and Alzheimer’s disease (AD). Furthermore, dietary supplementation with B vitamins, n-3 polyunsaturated fatty acids, and polyphenols may enhance brain health [239].

A growing body of evidence supports the notion that diet can improve symptoms associated with a wide range of neurological disorders. The impact of nutrition on disease risk and treatment response in conditions such as PD, AD, stroke, Multiple Sclerosis, amyotrophic lateral sclerosis, and Huntington’s disease is shaped by processes like epigenetic modification, neuronal inflammation, and metabolic regulation [240].

Fasting, in particular, deserves a special mention. It has evolved alongside human development, closely aligning with our physiology and optimizing health [241]. Although most evidence comes from animal models, fasting may reduce brain damage, enhance recovery after a stroke, improve cognition, boost bioenergetic response, and promote neuronal plasticity and resilience, potentially countering multiple neurological disorders, including brain cancer. It also has positive effects on chemotherapy responses.

## 13. Practical Implications, Future Lines, and Limitations

The findings presented in this review have significant practical implications, particularly in public health, clinical interventions, and research development. Regular physical activity emerges as a powerful non-pharmacological intervention to modulate cellular metabolism and manage neurological diseases such as Alzheimer’s, Parkinson’s, and Multiple Sclerosis. Given the central role of mitochondrial dysfunction in these diseases, exercise protocols that enhance mitochondrial biogenesis and reduce oxidative stress should be prioritized in therapeutic strategies. These interventions have the potential not only to delay disease progression but also to reduce symptoms and significantly improve the quality of life for affected individuals.

### 13.1. Limitations

The limitations of this review include the reliance on studies published only in English, potentially omitting relevant research in other languages, and a focus primarily on high-quality, peer-reviewed journal articles, excluding non-peer-reviewed sources like dissertations and conference proceedings. Additionally, while this review concentrated on the literature from 2015 to 2024, some seminal research outside this period may have been overlooked, possibly affecting the comprehensiveness of historical data on exercise and mitochondrial function in neurodegenerative diseases.

### 13.2. Future Research

Future research should focus on refining exercise prescriptions tailored to the specific metabolic needs of patients with neurological diseases. This research focus includes identifying exercise types, intensities, and durations that maximize benefits for mitochondrial function and overall cellular health. Moreover, advancements in biomarker development could help monitor mitochondrial function and oxidative stress levels, offering a more personalized treatment approach. Additionally, integrating nutritional strategies with physical activity could amplify therapeutic outcomes by addressing metabolic disturbances comprehensively.

### 13.3. Practical Applications

- Development of Evidence-Based Public Health Campaigns: It is imperative to initiate and promote scientifically grounded public health campaigns that emphasize the role of regular physical activity in mitigating the progression of neurodegenerative diseases. Emerging evidence suggests that physical activity, through its positive effects on mitochondrial biogenesis and antioxidant defense mechanisms, can modulate key metabolic pathways involved in the pathophysiology of diseases like Alzheimer’s and Parkinson’s. Such campaigns should focus on increasing public awareness of these benefits, targeting populations at higher risk due to genetic predispositions or sedentary lifestyles, and should be grounded in data demonstrating the protective effects of exercise on neural plasticity and cognitive function.
- Implementation of Targeted Community-Based Exercise Programs: Community-level interventions that incorporate structured and supervised exercise programs for individuals at risk of, or already diagnosed with, neurodegenerative diseases should be a public health priority. These programs must be tailored to meet the specific metabolic and physical needs of these populations, drawing on the principles of personalized medicine. Evidence from randomized controlled trials (RCTs) suggests that regular aerobic exercise and resistance training can significantly improve motor function, cognitive capacity, and overall quality of life in patients with neurodegenerative diseases. These programs should not only aim to delay disease progression but also enhance neuroprotective mechanisms through improved mitochondrial dynamics and cellular resilience.
- Policy Development for Healthy Lifestyles and Mitochondrial Health: There is an urgent need for public health policies that incentivize the adoption of lifestyle modifications aimed at enhancing mitochondrial health. These policies should encourage both physical activity and nutritional interventions, which have been shown to reduce oxidative stress and improve mitochondrial function in patients with neurodegenerative diseases. Government and healthcare institutions must collaborate to create environments conducive to these lifestyle changes, including access to fitness facilities, subsidies for nutritional supplements, and education on the benefits of maintaining mitochondrial integrity through diet and exercise.
- Personalized Exercise Protocols Based on Metabolic and Clinical Needs: Collaborative efforts between healthcare providers and exercise physiologists are essential in developing personalized exercise regimens. These regimens should be customized according to an individual’s metabolic requirements, disease progression, and physical capacity, taking into account the patient’s mitochondrial function and oxidative stress levels. Recent studies have demonstrated that tailoring exercise programs to meet these specific needs can maximize the therapeutic outcomes for neurodegenerative disease patients, leading to improvements in both motor and cognitive function. Precision medicine approaches in this context could leverage biomarkers to optimize the intensity, frequency, and duration of physical activity for each patient.
- Integration of Exercise into Multimodal Rehabilitation Programs: The incorporation of physical activity into multimodal rehabilitation programs should be prioritized for patients with Alzheimer’s, Parkinson’s, and Multiple Sclerosis. Exercise has been shown to not only improve cardiovascular health but also enhance neurogenesis, synaptic plasticity, and mitochondrial function, thereby addressing both the physical and cognitive deficits associated with these diseases. Multidisciplinary approaches that combine physiotherapy, occupational therapy, and structured exercise have demonstrated significant improvements in patients’ functional capacities, particularly when initiated during the early stages of disease progression.
- Early-Life Preventive Exercise Strategies: There is substantial evidence to support the early implementation of physical activity programs as a preventive measure against the onset of neurodegenerative diseases. Studies indicate that individuals who engage in regular physical activity from an early age exhibit enhanced mitochondrial efficiency and greater resistance to oxidative stress, both of which are crucial in preventing the cellular and metabolic impairments that contribute to neurodegeneration. Public health initiatives should focus on instilling these exercise habits in younger populations, with the long-term goal of reducing the incidence and severity of age-related neurodegenerative diseases.

These comprehensive, evidence-based strategies are crucial for maximizing the effectiveness of non-pharmacological interventions in maintaining mitochondrial function, delaying the progression of neurodegenerative diseases, and improving the overall quality of life for individuals at risk. By integrating exercise into preventive and therapeutic frameworks, public health efforts can address the metabolic underpinnings of these conditions and offer a holistic approach to managing and potentially preventing neurological disease.

## 14. Conclusions

The findings of this review not only underscore the critical role of exercise in cellular metabolism and the management of neurodegenerative conditions such as multiple sclerosis, Parkinson’s disease, and Alzheimer’s disease but also highlight its significance in promoting healthy aging. Aging is intrinsically linked to declines in mitochondrial function, increased oxidative stress, and cumulative cellular damage, all of which contribute to age-related diseases and functional decline. By enhancing mitochondrial biogenesis, reducing oxidative damage, and improving overall cellular health, regular physical activity serves as a cornerstone for slowing the aging process and maintaining physiological resilience.

Customized exercise programs tailored to the metabolic needs of individuals, particularly older adults, can play a transformative role in mitigating the effects of aging and reducing the risk of developing age-associated neurodegenerative diseases. When combined with targeted nutritional interventions, these programs have the potential to optimize therapeutic outcomes by addressing metabolic disturbances comprehensively.

Integrating exercise and dietary strategies into both preventive and therapeutic frameworks offers a holistic approach to managing aging-related health challenges. This dual approach not only enhances the quality of life for older adults but also supports healthy longevity by preserving cognitive function, physical independence, and metabolic homeostasis.

Future research should prioritize the development of precise exercise protocols, exploring the most effective types, intensities, and durations of activity to combat age-related cellular decline. Additionally, advancements in biomarker technology for assessing mitochondrial health and oxidative stress will be pivotal in creating personalized interventions that maximize the benefits of exercise and nutrition for healthy aging. By emphasizing these lifestyle-based interventions, healthcare practices can better equip individuals to age gracefully while minimizing the burden of chronic diseases.

## Data Availability

Not applicable.
